# The Taiwan Precision Medicine Initiative provides a cohort for large-scale studies

**DOI:** 10.1038/s41586-025-09680-x

**Published:** 2025-10-15

**Authors:** Hsin-Chou Yang, Pui-Yan Kwok, Ling-Hui Li, Yi-Min Liu, Yuh-Jyh Jong, Kang-Yun Lee, Da-Wei Wang, Ming-Fang Tsai, Jenn-Hwai Yang, Chien-Hsiun Chen, Erh-Chan Yeh, Chun-yu Wei, Cathy S.-J. Fann, Yen-Tsung Huang, Chia-Wei Chen, Yi-Ju Lee, Shih-Kai Chu, Chih-hsing Ho, Cheng-Shin Yang, Yungling Leo Lee, Hung-Hsin Chen, Ming-Chih Hou, Jeng-Fong Chiou, Shun-Fa Yang, Chih-Hung Wang, Chih-Yang Huang, Kuan-Ming Chiu, Ming Chen, Fu-Tien Chiang, Sing-Lian Lee, Shiou-Sheng Chen, Wei-Jen Yao, Chih-Cheng Chien, Shih-Yao Lin, Fu-Pang Chang, Hsiang-Ling Ho, Yi-Chen Yeh, Wei-Cheng Tseng, Ming-Hwai Lin, Hsiao-Ting Chang, Ling-Ming Tseng, Wen-Yih Liang, Paul Chih-Hsueh Chen, Jen-Fan Hang, Shih-Chieh Lin, Yu-Jiun Chan, Ying-Ju Kuo, Lei-Chi Wang, Chin-Chen Pan, Yu-Cheng Hsieh, Yi-Ming Chen, Tzu-Hung Hsiao, Ching-Heng Lin, Yen-Ju Chen, I-Chieh Chen, Chien-Lin Mao, Shu-Jung Chang, Yen-Lin Chang, Yi-Ju Liao, Chih-Hung Lai, Wei-Ju Lee, Hsin Tung, Ting-Ting Yen, Hsin-Chien Yen, Chun-Ming Shih, Teh-Ying Chou, Tsan-Hon Liou, Chen-Yuan Chiang, Yih-Giun Cherng, Chih-Hwa Chen, Chao-Hua Chiu, Sung-Hui Tseng, Emily Pei-Ying Lin, Ying-Ju Chen, Hui-Ping Chuang, Tsai-Chuan Chen, Wei-Ting Huang, Joey Sin, I-Ling Liu, Yi-Chen Chen, Kuo-Kuang Chao, Yu-Min Wu, Pin-Pin Yu, Lung-Pao Chang, Kuei-Yao Yen, Li-Ching Chang, Yi-Jing Sheen, Yuan-Tsong Chen, Kamhon Kan, Hsiang-Lin Tsai, Yao-Kuang Wang, Ming-Feng Hou, Yuan-Han Yang, Chao-Hung Kuo, Wen-Jeng Wu, Jee-Fu Huang, Inn-Wen Chong, Jong-Rung Tsai, Cheng-Yu Lin, Ming-Chin Yu, Tsong-Hai Lee, Meng-Han Tsai, Yu-Che Ou, Pin-Yuan Chen, Tsung-Hui Hu, Yu-Chiau Shyu, Chih-Kuang Cheng, Yu-Jen Fang, Song-Chou Hsieh, Chien-Hung Chen, Chieh-Chang Chen, Ko-Jen Li, Chin-Hsien Lin, Hsien-Yi Chiu, Chen-Chi Wu, Chun-Yen Chen, Shi-Jye Chu, Feng-Cheng Liu, Fu-Chi Yang, Hsin-An Chang, Wei-liang Chen, Sung-Sen Yang, Yueh-feng Sung, Tso-Fu Wang, Shinn-Zong Lin, Yen-Wen Wu, Chien-Sheng Wu, Ju-Ying Jiang, Gwo-Chin Ma, Ting-Yu Chang, Juey-Jen Hwang, Kuo-Jang Kao, Chen-Fang Hung, Ting-Fang Chiu, Po-Yueh Chen, Kochung Tsui, Ming-Shiang Wu, See-Tong Pang, Shih-Ann Chen, Wei-Ming Chen, Chun-houh Chen, Wayne Huey-Herng Sheu, Jer-Yuarn Wu

**Affiliations:** 1 Institute of Statistical Science, Academia Sinica, Taipei, Taiwan; 2 Biomedical Translation Research Center, Academia Sinica, Taipei, Taiwan; 3 Institute of Biomedical Sciences, Academia Sinica, Taipei, Taiwan; 4 Cardiovascular Research Institute, Institute for Human Genetics, and Department of Dermatology, University of California, San Francisco, CA USA; 5 Graduate Institute of Clinical Medicine, Kaohsiung Medical University, Kaohsiung, Taiwan; 6 Departments of Pediatrics and Laboratory Medicine, Kaohsiung Medical University Hospital, Kaohsiung, Taiwan; 7 Department of Biological Science and Technology, National Yang Ming Chiao Tung University, Hsinchu, Taiwan; 8 Department of Internal Medicine, School of Medicine, College of Medicine, Taipei Medical University, Taipei, Taiwan; 9 Division of Thoracic Medicine, Department of Internal Medicine, Taipei Medical University Shuang Ho Hospital, Taipei, Taiwan; 10 Institute of Information Sciences, Academia Sinica, Taipei, Taiwan; 11 Core Laboratory of Neoantigen Analysis for Personalized Cancer Vaccine, Office of R&D, Taipei Medical University, Taipei, Taiwan; 12 Bioinformatics Program, Taiwan International Graduate Program, Academia Sinica, Taipei, Taiwan; 13 Department of Mathematics, Institute of Epidemiology and Preventive Medicine, National Taiwan University, Taipei, Taiwan; 14 Department of Statistics, National Taipei University, Taipei, Taiwan; 15 Institute of European and American Studies, Academia Sinica, Taipei, Taiwan; 16 Division of Gastroenterology and Hepatology, Department of Medicine, Taipei Veterans General Hospital, Taipei, Taiwan; 17 Department of Radiology, School of Medicine, College of Medicine, Taipei Medical University, Taipei, Taiwan; 18 Department of Radiation Oncology, Taipei Medical University Hospital, Taipei, Taiwan; 19 Institute of Medicine, Chung Shan Medical University, Taichung, Taiwan; 20 Department of Medical Research, Chung Shan Medical University Hospital, Taichung, Taiwan; 21 Tri-Service General Hospital, National Defense Medical Center, Taipei, Taiwan; 22 Taipei Veterans General Hospital, Taoyuan Branch, Taoyuan, Taiwan; 23 Cardiovascular and Mitochondrial Related Disease Research Center, Hualien Tzu Chi Hospital, Buddhist Tzu Chi Medical Foundation, Hualien, Taiwan; 24 Center of General Education, Buddhist Tzu Chi Medical Foundation, Tzu Chi University of Science and Technology, Hualien, Taiwan; 25 Far Eastern Memorial Hospital, New Taipei City, Taiwan; 26 Department of Electrical Engineering, Yuan Ze University, Taoyuan, Taiwan; 27 Department of Genomic Medicine and Center for Medical Genetics, Changhua Christian Hospital, Changhua, Taiwan; 28 Department of Cardiology, Fu Jen Catholic University Hospital, Fu Jen Catholic University, New Taipei City, Taiwan; 29 School of Medicine, College of Medicine, Fu Jen Catholic University, New Taipei City, Taiwan; 30 Department of Internal Medicine, Koo Foundation Sun Yat-Sen Cancer Center, Taipei, Taiwan; 31 Division of Urology, Taipei City Hospital, Ren Ai Branch, Taipei, Taiwan; 32 General Education Center, University of Taipei, Taipei, Taiwan; 33 Department of Urology, College of Medicine and Shu-Tien Urological Research Center, National Yang-Ming Chiao Tung University, Taipei, Taiwan; 34 Department of Nuclear Medicine, Ditmanson Medical Foundation Chia-Yi Christian Hospital, Chiayi City, Taiwan; 35 School of Medicine, Fu-Jen Catholic University, New Taipei City, Taiwan; 36 Department of Anesthesiology, Cathay General Hospital, Taipei, Taiwan; 37 Department of Pathology and Laboratory Medicine, Taipei Veterans General Hospital, Taipei, Taiwan; 38 School of Medicine, National Yang Ming Chiao Tung University, Taipei, Taiwan; 39 Department of Biotechnology and Laboratory Science in Medicine, National Yang Ming Chiao Tung University, Taipei, Taiwan; 40 Division of Nephrology, Department of Medicine, Taipei Veterans General Hospital, Taipei, Taiwan; 41 School of Medicine, College of Medicine, National Yang Ming Chiao Tung University, Taipei, Taiwan; 42 Center for Intelligent Drug Systems and Smart Bio-devices, National Yang Ming Chiao Tung University, Hsinchu, Taiwan; 43 Department of Family Medicine, Taipei Veterans General Hospital, Taipei, Taiwan; 44 Division of General Surgery, Department of Surgery, Taipei Veterans General Hospital, Taipei, Taiwan; 45 Comprehensive Breast Health Center, Taipei Veterans General Hospital, Taipei, Taiwan; 46 Institute of Clinical Medicine, National Yang Ming Chiao Tung University, Taipei, Taiwan; 47 Center for Infection Control, Taipei Veterans General Hospital, Taipei, Taiwan; 48 Institute of Public Health, National Yang Ming Chiao Tung University, Taipei, Taiwan; 49 Department of Medical Research, Taichung Veterans General Hospital, Taichung, Taiwan; 50 Department of Post-Baccalaureate Medicine, National Chung Hsing University, Taichung, Taiwan; 51 Department of Pharmacy, Taichung Veterans General Hospital, Taichung, Taiwan; 52 Department of Medicine and Cardiovascular Center, Taichung Veterans General Hospital, Taichung, Taiwan; 53 Neurological Institute, Taichung Veterans General Hospital, Taichung, Taiwan; 54 Department of Otolaryngology, Taichung Veterans General Hospital, Taichung, Taiwan; 55 Division of Pediatric Genetics and Metabolism, Children’s Medical Center, Taichung Veterans General Hospital, Taichung, Taiwan; 56 Cardiovascular Research Center, Taipei Medical University Hospital, Taipei, Taiwan; 57 Taipei Heart Institute, Taipei Medical University, Taipei, Taiwan; 58 Graduate Institute of Clinical Medicine, School of Medicine, Taipei Medical University, Taipei, Taiwan; 59 Department of Pathology and Precision Medicine Research Center, Taipei Medical University Hospital, Taipei Medical University, Taipei, Taiwan; 60 Department of Physical Medicine and Rehabilitation, School of Medicine, College of Medicine, Taipei Medical University, Taipei, Taiwan; 61 Division of Rehabilitation Medicine, Taipei Municipal Wanfang Hospital, Taipei Medical University, Taipei, Taiwan; 62 Division of Pulmonary Medicine, Department of Internal Medicine, School of Medicine, College of Medicine, Taipei Medical University, Taipei, Taiwan; 63 Division of Pulmonary Medicine, Department of Internal Medicine, Wan Fang Hospital, Taipei Medical University, Taipei, Taiwan; 64 Department of Anesthesiology, School of Medicine, College of Medicine, Taipei Medical University, Taipei, Taiwan; 65 Division of Anesthesiology, Taipei Medical University Shuang Ho Hospital, Taipei, Taiwan; 66 Department of Orthopedics, Shuang Ho Hospital, Taipei Medical University, Taipei, Taiwan; 67 School of Biomedical Engineering, College of Biomedical Engineering, Taipei Medical University, Taipei, Taiwan; 68 School of Medicine, College of Medicine, Taipei Medical University, Taipei, Taiwan; 69 Division of Thoracic Medicine, Department of Internal Medicine, Taipei Medical University Hospital, Taipei, Taiwan; 70 Division of Physical Medicine and Rehabilitation, Taipei Medical University Hospital, Taipei, Taiwan; 71 Department of Thoracic Medicine, Department of Internal Medicine, School of Medicine, College of Medicine, Taipei Medical University, Taipei, Taiwan; 72 Precision Medicine Research Center, Taipei Medical University Hospital, Taipei, Taiwan; 73 Division of Endocrinology and Metabolism, Department of Internal Medicine, Taichung Veterans General Hospital, Taichung, Taiwan; 74 Department of Medicine, School of Medicine, National Yang Ming Chiao Tung University, Taipei, Taiwan; 75 Department of Post-Baccalaureate Medicine, College of Medicine, National Chung Hsing University, Taichung, Taiwan; 76 Institute of Economics, Academia Sinica, Taipei, Taiwan; 77 Division of Colorectal Surgery, Department of Surgery, Kaohsiung Medical University Hospital, Kaohsiung Medical University, Kaohsiung, Taiwan; 78 Division of Gastroenterology, Department of Internal Medicine, Kaohsiung Medical University Hospital, Kaohsiung, Taiwan; 79 Department of Medicine, Faculty of Medicine, College of Medicine, Kaohsiung Medical University, Kaohsiung, Taiwan; 80 Division of Breast Oncology and Surgery, Department of Surgery, Kaohsiung Medical University Chung-Ho Memorial Hospital, Kaohsiung, Taiwan; 81 Department of Neurology, Kaohsiung Municipal Ta-Tung Hospital, Kaohsiung Medical University, Kaohsiung, Taiwan; 82 Division of Gastroenterology, Department of Internal Medicine, Kaohsiung Municipal Siaogang Hospital, Kaohsiung, Taiwan; 83 Department of Urology, Kaohsiung Medical University Chung-Ho Memorial Hospital, Kaohsiung Medical University, Kaohsiung, Taiwan; 84 Hepatobiliary Division, Department of Internal Medicine, Kaohsiung Medical University Hospital, Kaohsiung Medical University, Kaohsiung, Taiwan; 85 College of Medicine, Kaohsiung Medical University, Kaohsiung, Taiwan; 86 Hepatitis Center, Kaohsiung Medical University Hospital, Kaohsiung Medical University, Kaohsiung, Taiwan; 87 Division of Pulmonary and Critical Care Medicine, Department of Internal Medicine, Kaohsiung Medical University Hospital, Kaohsiung Medical University, Kaohsiung, Taiwan; 88 Department of Internal Medicine, Kaohsiung Municipal Cijin Hospital, Kaohsiung, Taiwan; 89 Division of Respiratory Therapy, College of Medicine, Kaohsiung Medical University, Kaohsiung, Taiwan; 90 Department of Gastroenterology and Hepatology, Linkou Chang Gung Memorial Hospital, Taoyuan, Taiwan; 91 Department of Surgery, New Taipei Municipal TuCheng Hospital (built and operated by Chang Gung Medical Foundation) and Chang Gung University, New Taipei City, Taiwan; 92 Department of Neurology and Stroke Center, Linkou Chang Gung Memorial Hospital, Taoyuan, Taiwan; 93 College of Medicine, Chang Gung University, Taoyuan, Taiwan; 94 School of Medicine, College of Medicine, Chang Gung University, Taoyuan, Taiwan; 95 Department of Neurology, Kaohsiung Chang Gung Memorial Hospital, Kaohsiung, Taiwan; 96 Department of Obstetrics and Gynecology, Chiayi Chang Gung Memorial Hospital, Chiayi, Taiwan; 97 Department of Neurosurgery, Chang Gung Memorial Hospital, Keelung Branch, Keelung, Taiwan; 98 Division of Hepato-Gastroenterology, Department of Internal Medicine, Kaohsiung Chang Gung Memorial Hospital, Kaohsiung, Taiwan; 99 Chang Gung University College of Medicine, Kaohsiung, Taiwan; 100 Community Medicine Research Center, Chang Gung Memorial Hospital, Keelung Branch, Keelung, Taiwan; 101 Bachelor Degree Program in Marine Biotechnology, National Taiwan Ocean University, Keelung, Taiwan; 102 Department of Neurology, Chang Gung Memorial Hospital, Taipei, Taiwan; 103 Department of Internal Medicine, National Taiwan University College of Medicine, Taipei, Taiwan; 104 Department of Internal Medicine, National Taiwan University Hospital, Yun-Lin Branch, National Taiwan University College of Medicine, Yun-Lin, Taiwan; 105 Division of Rheumatology, Immunology and Allergy, Department of Internal Medicine, National Taiwan University Hospital, National Taiwan University College of Medicine, Taipei, Taiwan; 106 Division of Gastroenterology and Hepatology, Department of Internal Medicine, National Taiwan University Hospital, Taipei, Taiwan; 107 Department of Internal Medicine, National Taiwan University Cancer Center, Taipei, Taiwan; 108 Graduate Institute of Clinical Medicine, College of Medicine, National Taiwan University, Taipei, Taiwan; 109 Division of Gastroenterology and Hepatology, Department of Internal Medicine, National Taiwan University Hospital, College of Medicine, National Taiwan University, Taipei, Taiwan; 110 Department of Neurology, National Taiwan University Hospital, College of Medicine, National Taiwan University, Taipei, Taiwan; 111 Department of Medical Research, National Taiwan University Hospital, Hsin-Chu Branch, Hsinchu, Taiwan; 112 Psychiatry, Tri-Service General Hospital, Taipei, Taiwan; 113 Rheumatology, Immunology and Allergy, Tri-Service General Hospital, Taipei, Taiwan; 114 Neurology, Tri-Service General Hospital, Taipei, Taiwan; 115 Family Medicine, Tri-Service General Hospital, Taipei, Taiwan; 116 Nephrology, Tri-Service General Hospital, Taipei, Taiwan; 117 Department of Hematology and Oncology, Hualien Tzu Chi Hospital, Buddhist Tzu Chi Medical Foundation, Hualien, Taiwan; 118 School of Medicine, Tzu Chi University, Hualien, Taiwan; 119 Department of Neurosurgery, Hualien Tzu Chi Hospital, Hualien, Taiwan; 120 Integration Center of Traditional Chinese and Modern Medicine, Hualien Tzu Chi Hospital, Buddhist Tzu Chi Medical Foundation, Hualien, Taiwan; 121 Division of Cardiology, Cardiovascular Medical Center, Far Eastern Memorial Hospital, New Taipei City, Taiwan; 122 Graduate Institute of Medicine, Yuan Ze University, Taoyuan, Taiwan; 123 Division of Allergy, Immunology and Rheumatology, Far Eastern Memorial Hospital, New Taipei City, Taiwan; 124 School of Medicine, Fu Jen Catholic University, New Taipei City, Taiwan; 125 Division of Endocrinology and Metabolism, Department of Medicine, Far Eastern Memorial Hospital, New Taipei City, Taiwan; 126 Koo Foundation Sun Yat-Sen Cancer Center, Taipei, Taiwan; 127 Department of Pediatrics, Taipei City Hospital, Zhongxiao Branch, Taipei, Taiwan; 128 Department of Health and Welfare, University of Taipei, Taipei, Taiwan; 129 Graduate Institute of Business Administration, Fu Jen Catholic University, New Taipei City, Taiwan; 130 Division of Gastroenterology and Hepatology, Department of Internal Medicine, Ditmanson Medical Foundation Chia-Yi Christian Hospital, Chiayi City, Taiwan; 131 Clinical Trial Center, Department of Medical Research, Ditmanson Medical Foundation Chia-Yi Christian Hospital, Chiayi City, Taiwan; 132 Cathay General Hospital Department of Clinical Pathology, Taipei, Taiwan; 133 Cathay General Hospital Department of Internal Medicine, Taipei, Taiwan; 134 Division of Urology, Department of Surgery, Chang Gung Memorial Hospital, Linkou Branch, Taoyuan, Taiwan; 135 Cardiovascular Center, Taichung Veterans General Hospital, Taichung, Taiwan; 136 Heart Rhythm Center, Division of Cardiology, Department of Medicine, Taipei Veterans General Hospital, Taipei, Taiwan; 137 Department of Orthopaedics and Traumatology, Taipei Veterans General Hospital, Taipei, Taiwan; 138 Institute of Molecular and Genomic Medicine, National Health Research Institutes, Zhunan, Taiwan; 139 Division of Endocrinology and Metabolism, Department of Internal Medicine, Taipei Veterans General Hospital, Taipei, Taiwan

**Keywords:** Genetic markers, Genome-wide association studies, Medical genetics, Genetic predisposition to disease, Health care

## Abstract

Han Chinese people comprise nearly 20% of the global population but remain under-represented in genetic studies^1,2^, so there is an urgent need for large-scale cohorts to advance precision medicine. Here we present the Taiwan Precision Medicine Initiative (TPMI), established by Academia Sinica in collaboration with 16 major medical centres around Taiwan, which has recruited 565,390 participants who consent to provide DNA samples for genetic profiling and grant access to their electronic medical records (EMRs) for research. EMR access is both retrospective and prospective, allowing longitudinal studies. Genetic profiling is done with population-optimized arrays of single-nucleotide polymorphisms for people of Han Chinese ancestry, which enable genome-wide association^3,4^, phenome-wide association^5,6^ and polygenic risk score^7,8^ studies to be performed to evaluate common disease risk and pharmacogenetic response. Participants also agreed to be re-contacted for future research and receive personalized genetic risk profiles with health management recommendations. The TPMI has established the TPMI Data Access Platform, a central database and analysis platform that both safeguards the security of the data and facilitates academic research. As a large cohort of individuals with non-European ancestry that merges genetic profiles with EMR data and enables longitudinal follow-up, TPMI provides a unique resource that could be used to validate genetic risk prediction models, perform clinical trials of risk-based health management and inform health policies. Ultimately, the TPMI cohort will contribute to global genetic research and serve as a model for population-based precision medicine.

## Abstract (web summary)

The Taiwan Precision Medicine Initiative (TPMI) has built a cohort of more than half a million individuals with Han Chinese ancestry, providing a rich resource of genetic and medical data for this group.

## Main

Precision medicine is a global movement to improve health outcomes by tailoring medical interventions to the unique characteristics of individual patients^9,10^. For this movement to succeed, large cohorts with known disease states and rich clinical data must be built so that they can be analysed against genetic and other factors that contribute to disease risk and treatment outcomes. Once established and validated, members of the population can match their own profiles against those from the study cohorts and identify the best medical management for them. In recent years, precision medicine initiatives around the world^11^ have enriched the research landscape by producing comprehensive datasets (consisting of demographic, genetic, biomedical and clinical, environmental and behavioural, lifestyle and food preference and contextual information) along with biospecimens from large cohorts. These invaluable resources offer the potential to advance disease prognosis prediction, risk assessment and medical and healthcare through personalized medicine and precision health. However, the vast majority of large studies are conducted in populations of European ancestry^12,13^, with results that are not optimal for use in other populations. The Taiwan Precision Medicine Initiative (TPMI) is designed to create a cohort of Han Chinese ancestry to address the needs of almost 20% of the world’s population.

The TPMI, a consortium established by Academia Sinica in collaboration with 16 partner medical centres across the country (Supplementary Fig. 1), has built a large cohort of participants who consent to provide DNA samples for genetic profiling and grant access to their EMRs for studies to develop precision medicine. Of note, EMR access is both retrospective and prospective, ensuring that longitudinal follow-up of each participant is possible. In return, genetic risk profiling results are shared with the participants, with an invitation to participate in follow-up studies to validate disease risk prediction models and risk-based healthcare management guidelines. Key components of the TPMI project include (a) recruitment of a large number of participants from medical facilities in Taiwan; (b) development of population-optimized single-nucleotide polymorphism (SNP) arrays; (c) establishment of a dedicated research database for genetic profiles and EMRs; (d) construction of a user-friendly data analysis platform and workplace to facilitate retrieval, summary and visualization of data for researchers; (e) analysis of genetic profiles and clinical data of the cohort, with a focus on creating algorithms for polygenic risk scores (PRSs) to assess common disease risk and pharmacogenetic response; and (f) active engagement in public education initiatives aimed at enhancing people’s understanding of genetics and precision medicine. The project timeline and milestones are shown in Supplementary Fig. 2 and Supplementary Data 1.

## Enrolment of participants

**Fig. 1:**
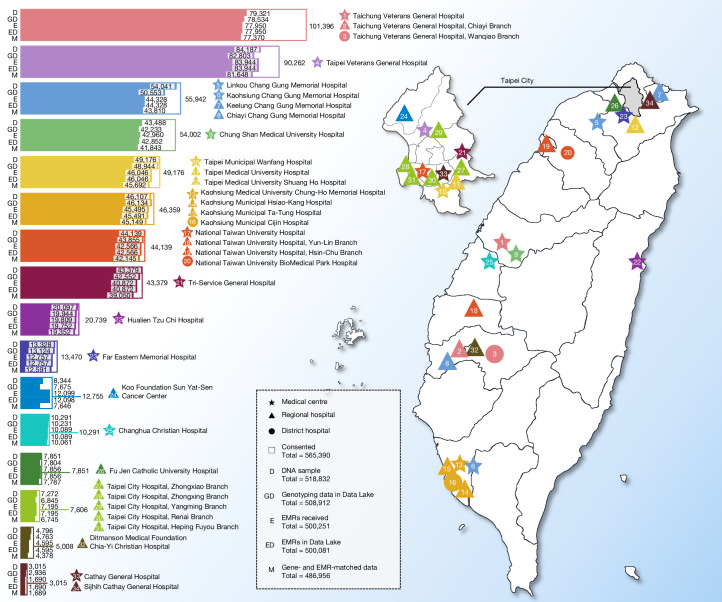
Map of medical centres, their satellite hospitals and sample sizes. Locations of 16 partner medical centres and 33 affiliated hospitals, along with the numbers of DNA samples, genotyped samples, individuals with EMRs received, individuals with EMRs stored in the TPMI Data Lake and individuals with both genotype and EMR data. Source Data.

To ensure compliance with local guidelines, we followed the Taiwan Ministry of Health and Welfare regulations for ethical approval, patient data protection and clinical research and care. Participants were recruited from 16 partner medical centres (encompassing 33 affiliated hospitals) that together serve around 40% of the population in Taiwan (Fig. 1). On-site physicians and nurses facilitated the enrolment process, mainly through outpatient departments. Informed consent was obtained from the participants while they were enrolled in this study at the hospitals (Supplementary Note 1). After providing informed consent, participants donated blood samples for genotyping and agreed to have their EMRs de-identified, encrypted and securely transmitted to the TPMI server. Genetic profiling by genotyping was done using two customized TPMI SNP arrays (TPMv1 and TPMv2; Supplementary Note 2), with SNP content (Supplementary Table 1), SNP minor allele frequency (MAF) (Supplementary Table 2) and shared SNPs between the two arrays (Supplementary Fig. 3 and Supplementary Data 2) are provided. Participation was offered to all except for those whose peripheral blood cells might contain non-germline genetic materials: (a) individuals with leukaemia who had not gone into remission; (b) individuals who had received blood transfusions within the previous six months; and (c) individuals who had undergone chemotherapy or radiotherapy within the previous 12 months. Although no intentional oversampling was conducted, some disease over-representation might exist, because TPMI partner hospitals are medical centres that typically serve patients with more chronic or severe conditions. The enrolment rate among invited individuals was approximately 60–80%. As of 28 December 2023 (TPMI v37 data freeze), 565,390 participants had been enrolled with proper consent.

## Genotyping and imputation

Genotyping assays were performed at the National Center for Genome Medicine in the Academia Sinica and six partner hospitals (see Methods, ‘Genotyping and plate normalization’). After quality-control measures, we have genotypes for TPMv1 in 99 batches, consisting of 165,596 individuals, and for TPMv2 in 114 batches, comprising 321,360 individuals (486,956 total) with matching EMR data (Version 37). To assess the performance of our phasing and imputation pipeline (see Methods, ‘Imputation’), 6,000 genotyped variants on chromosomes 5, 13 and 18, from 1,000 individuals in the TPMI, were randomly masked. We assessed imputation quality scores (INFO scores) and the correlation between imputed and observed genotypes. We found an average correlation of 0.906 for all masked variants, and 96.3% of the masked SNPs had an INFO score greater than 0.7. Compared with two sequencing-based Han Chinese datasets, ChinaMAP^14^ (*n* = 10,588) and the Westlake BioBank for Chinese (WBBC)^15^ (*n* = 4,480 after data quality control), the TPMI imputation dataset identified 388,545 novel variants (4.83%) not present in either resource (Supplementary Table 3). The large sample size of the TPMI enables robust detection of low-frequency variants with confidence. Moreover, its broad geographical coverage of subpopulations with Han Chinese ancestry (see ‘Population structure’ section below) facilitates the identification of novel variants that capture the genetic diversity and structure of the contemporary Taiwanese population.

## EMR data

To minimize the burden on the information technology staff at the partner hospitals, the TPMI adopted the strategy of taking EMR data from the hospitals ‘as is’, except with personal identifying information removed. The TPMI information technology team extracted and standardized the data from diverse hospital data formats into a searchable database to facilitate analysis. For each participant, data from five years before enrolment, as well as from subsequent hospital and clinic visits, were transmitted to the TPMI database (the TPMI Data Lake). A total of 250,000 participants have medical records of 5 years or more, and 73,000 of them have records of 10 years or more. The collected EMR data consist of outpatient records, discharge summaries, laboratory test results, pathology reports, surgery reports and imaging reports (Supplementary Fig. 4). Each type of record includes both free-text sections (for example, condition summaries in outpatient records; more details in Supplementary Table 4) and predefined structured data (for example, International Classification of Diseases (ICD) diagnosis based on ICD-9 or ICD-10 in outpatient records; more details in Supplementary Table 4). To deal with the many EMR data formats, the Academia Sinica information technology team implemented a series of data quality-control measures during the data import process. These measures include data cleaning, correction, standardization and extraction. After quality control, data were restructured and organized into a custom tabular format, improving search capabilities and overall usability. The team extracted pertinent information from free-text data using NLP models or regular expressions for further research analysis. For instance, spaCy models were developed to extract lifestyle data of participants, such as smoking, drinking and betel-nut consumption. At the same time, regular expressions were used to extract the results of cognitive tests, including the Mini-Mental Status Examination, the Cognitive Abilities Screening Instrument and the Clinical Dementia Rating. In total, 144 EMR variables have been catalogued in the TPMI Data Analysis Platform (TDAP) (Supplementary Note 3). Access to data is governed by guidelines found in Supplementary Note 4 and the Data availability statement.

## Cohort characteristics

**Fig. 2:**
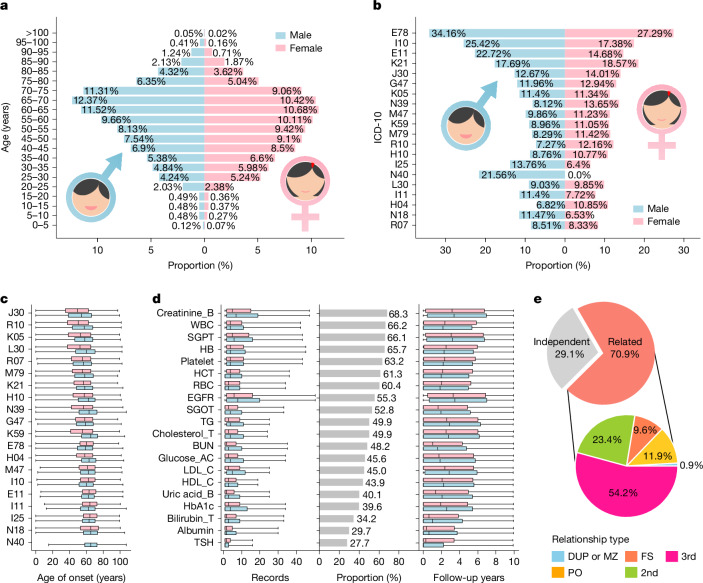
Cohort characteristics. **a**, Sex-specific age distribution. **b**, Top 20 most prevalent ICD-10 codes: E78 (disorders of lipoprotein metabolism and other lipidaemias), I10 (EHT), E11 (type 2 diabetes mellitus), K21 (gastro-oesophageal reflux disease), J30 (vasomotor and allergic rhinitis), G47 (sleep disorders), K05 (gingivitis and periodontal diseases), N39 (other urinary disorders), M47 (spondylosis), K59 (other functional intestinal disorders), M79 (other and unspecified soft tissue disorders), R10 (abdominal and pelvic pain), H10 (conjunctivitis), I25 (chronic ischaemic heart disease), N40 (enlarged prostate), L30 (other and unspecified dermatitis), I11 (hypertensive heart disease), H04 (lacrimal system disorders), N18 (chronic kidney disease) and R07 (pain in throat or chest). **c**, Age of onset for the top 20 diseases. Onset ages in male individuals (blue) and female individuals (pink) are presented as box plots, ordered by median. Box plots represent minima, first quartile, median, third quartile and maxima. Values and sample sizes are in the Source Data. **d**, Top 20 most prevalent laboratory tests: creatinine_B (blood creatinine), WBC (white blood cell count), SGPT (serum glutamic pyruvic transaminase or alanine aminotransferase; S-GPT/ALT), HB (haemoglobin), platelet (platelet count), HCT (haematocrit), RBC (red blood cell count), EGFR (estimated glomerular filtration rate), SGOT (serum glutamic–oxaloacetic transaminase or aspartate aminotransferase; S-GOT/AST), TG (triglyceride), cholesterol_T (Total Cholesterol), BUN (blood urea nitrogen), glucose_AC (fasting glucose), LDL_C (low-density lipoprotein cholesterol), HDL_C (high-density lipoprotein cholesterol), uric acid_B (blood uric acid), HbA1c (haemoglobin A1c), bilirubin_T (bilirubin, total value), albumin and TSH (thyroid-stimulating hormone, measured by enzyme immunoassay or luminescence immunoassay). Left, sex-specific distribution of record counts per individual (winsorized at the 95th percentile); middle, proportion of individuals with test data; right, distribution of average follow-up years. Box plots represent minima, first quartile, median, third quartile and maxima. Values and sample sizes are in the Source Data. **e**, The top pie chart shows the proportions of related and unrelated samples. The bottom pie chart shows relationship categories: duplicate (DUP) or monozygotic twin (MZ), parent–offspring (PO), full sibling (FS), second degree (2nd) and third degree (3rd). Source Data.

Among the 486,956 participants with both genotype and EMR data, there are 217,595 male participants (average age of 57.4, s.d. = 17.5) and 269,361 female participants (average age of 54.9, s.d. = 17.0). Most participants fall within the age range of 20 to 90 years, with more than 160 centenarians (Fig. 2a).

Among the participants with ICD-10 codes, the top five prevalent diseases are disorders of lipoprotein metabolism and other lipidaemias (E78, 30.3%), essential hypertension (EHT) (I10, 21.0%), type 2 diabetes (T2D) (E11, 18.3%), gastro-oesophageal reflux disease (K21, 18.2%) and vasomotor and allergic rhinitis (J30, 13.4%) (Fig. 2b). Noteworthy sex differences in the diagnosis proportion were observed among the top 20 diseases, with exceptions noted in gingivitis and periodontal diseases (K05) (*P* = 0.56). The data suggest that adjustment for age and sex is necessary in subsequent genetic association analyses. These top 20 prevalent diseases have differential ages of onset: vasomotor and allergic rhinitis (J30) has the youngest average onset age (49.4 years; s.d. = 18.4), and enlarged prostate (N40) has the oldest (65.5 years; s.d. = 11.1) (Fig. 2c).

Laboratory test data show that 68.3% of the 486,956 participants have creatinine test records (averaging 5 test records per person, with an average follow-up duration of 3 years) (Fig. 2d). The second to fifth most frequently obtained laboratory tests are white blood cell count (66.2%), serum glutamic pyruvic transaminase (66.1%), haemoglobin (65.7%) and platelet count (63.2%) (Fig. 2d, left). These tests are integral components of standard diagnostic and preventive care panels, and their data availability may also be linked to prevalent health issues and chronic conditions. For instance, blood creatinine level to assess kidney function is also used to monitor urinary tract issues, hypertension or diabetes, conditions that are prevalent in Taiwan.

Familial relatedness analysis using kinship coefficient and identity by descent (see Methods, ‘Familial relatedness analysis’) reveals that 70.9% of participants can identify their third-degree or closer relatives among other TPMI participants, with the distribution of different levels of relatedness provided (Fig. 2e). For genetic association analysis, which assumes sample independence, it is necessary to select unrelated representative samples from each family. However, this approach results in a substantial decrease in sample size. Alternatively, a generalized mixed-effect approach, which analyses sample correlation by considering a random effect—such as SAIGE^16^ and REGENIE^17^ for case–control studies and BOLT-LMM^18^ and REGENIE^17^ for quantitative trait studies—can be used for genome-wide association studies (GWASs) without a reduction in sample size.

## Population structure

**Fig. 3:**
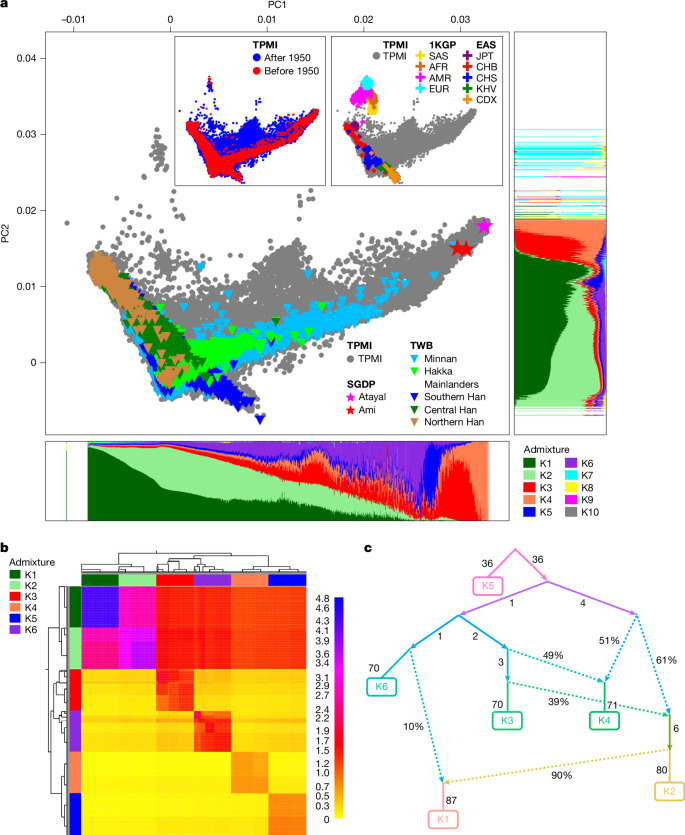
Population structure. **a**, PCA analysis. The TPMI cohort was compared with TWB, SGDP and 1KGP samples. The top-left inset compares TPMI participants born before 1950 with those born after 1950; the top-right inset compares the TPMI and the 1KGP. The main figure shows TPMI, TWB and two Taiwan Indigenous tribes (SGDP). Admixture fraction plots show ancestry fractions from ten ancestral populations (*K* = 10), with principal component (PC) 1 on the bottom axis and PC2 on the right. **b**, Coancestry and fine-scale structure. The coancestry heat map shows individuals (rows, columns) clustered by shared haplotypes, with colour intensity indicating haplotype copying. Darker blue or red indicates higher coancestry; yellow or light orange indicates lower. Diagonal blocks mark within-group sharing: K1–K6 show strong within-group haplotype sharing; K1–K2 (Han Chinese-enriched) exhibit strong coancestry with each other but less with K3–K6 (Indigenous-enriched), reflecting genetic differentiation; K3–K6 form distinct blocks, with some asymmetric sharing suggesting admixture or shared ancestry. The dendrogram shows clustering consistent with subgroup distinctions. **c**, Admixture graph depicting relationships and gene flow among K1–K6. Solid arrows represent drift edges (genetic drift from ancestral populations); dotted arrows represent admixture, with percentages indicating fractions. Edge numbers denote drift lengths (f2 units). K1 derives around 90% of ancestry from a lineage that also contributes to K2, plus 10% admixture from a lineage related to K6, indicating close K1–K2 affinity with minor Indigenous input. K4 shows around 49% of ancestry from a K5-related lineage (shared with K3) and 51% from a branch that also contributes to K2, reflecting Han–Indigenous admixture. K6 is mostly unadmixed with a long drift branch (f2 = 70), consistent with a highly diverged Indigenous lineage. K5 seems to be ancestral to other Indigenous groups (K3, K4 and possibly indirectly K6), with considerable early divergence (drift = 36 on both edges).

The population structure of the TPMI cohort was analysed against external resources with known population information, including the Taiwan Biobank (TWB)^19,20^, the Simons Genome Diversity Project (SGDP)^21^ and the 1000 Genomes Project (1KGP)^22^ (see Methods, ‘Population structure analysis’). Given the major influx of people from mainland China to Taiwan around 1950 (refs. ^23,24^), a separate principal component analysis (PCA) was conducted specifically for TPMI participants born before 1950, referred to as ‘<1950’, with a sample size of *n* = 70,708 (14.6%). The first two principal components (PCs) were used to construct a reference coordinate system, and subsequently, all other participants were projected onto the reference coordinate system (Fig. 3a).

TPMI participants born after 1950 exhibit a more diverse distribution, reflecting the historical intermarriage and genetic admixture between the major ethnic groups (the Minnan, the Hakka, and the Mainlanders) and minor Indigenous groups (Fig. 3a). It is worth noting that the TPMI and TWB (another large cohort project in Taiwan with a community-based design) have similar patterns in the PCA plot because the majority of both cohorts are of Han Chinese ancestry. However, the TPMI provides additional insights into the population structure of Indigenous groups (high PC1 and medium PC2) and the admixture between Han Chinese and Indigenous groups (medium PC1 and medium PC2), whereas TWB does not, because TWB data on Indigenous groups have not so far been released and analysed. Given the strong concordance between Han Chinese individuals in the TPMI and TWB in PCA space, along with previous evidence that TWB captures the full spectrum of Han Chinese genetic diversity^25^, the TPMI likewise serves as a comprehensive and representative resource for Han Chinese populations.

The first PC distinguishes mainly between the Han Chinese and the Indigenous groups. This is supported by comparing the TPMI data against data from individuals from two Indigenous tribes (Ami and Atayal) in the SGDP dataset (Fig. 3a) and integrative information from genetic admixture analysis (see below for details), recruitment hospital locations and government demographic statistics from the Council of Indigenous Peoples (https://www.cip.gov.tw/en/index.html) and the Department of Household Registration in Taiwan’s Ministry of the Interior (https://www.ris.gov.tw/app/en) (Supplementary Fig. 5 and Supplementary Data 3).

The second PC is correlated with latitude, in which higher PC2 scores correspond to northern latitudes and lower scores to southern latitudes. This pattern is observed in the TWB dataset (Southern, Central and Northern Han individuals from mainland China) and also in the 1KGP dataset (East Asian (EAS) individuals, including Chinese Dai in Xishuangbanna, China (CDX), Kinh in Ho Chi Minh City, Vietnam (KHV), Southern Han Chinese, China (CHS), Han Chinese in Beijing, China (CHB) and Japanese in Tokyo, Japan (JPT)). PCA and population admixture analysis allow us to adjust for population structure when conducting genetic studies and to exclude non-Han Chinese individuals from subsequent GWASs.

Genetic admixture analysis supported *K* = 10 as the optimal number of ancestral populations, on the basis of cross-validation error, log-likelihood stabilization and pairwise *F*_ST_ differentiation (see Methods, ‘Population structure analysis’, Supplementary Fig. 6a–c, Supplementary Data 4–6 and Supplementary Table 5). The ancestry fractions of TPMI participants were estimated and visualized along PC1 (Fig. 3a, bottom) and PC2 (Fig. 3a, right). Evidence from PCA analysis, genetic admixture analysis, recruitment hospital locations and government demographic statistics from the Council of Indigenous Peoples (https://www.cip.gov.tw/en/index.html) and the Department of Household Registration in Taiwan’s Ministry of the Interior (https://www.ris.gov.tw/app/en) guided the informative assignment of K1–K10 as follows: K1 represents North-enriched admixed Han (low PC1 and medium PC2) and K2 represents South-enriched admixed Han (low PC1 and low PC2). K3–K6 represent Taiwan’s Indigenous-enriched admixed groups (high PC1 and medium PC2). K7–K10 represent global immigrants (high PC2), with specific ancestry assignments: K7 corresponds to European (EUR) ancestry, K8 to South Asian (SAS) ancestry, K9 to American (AMR) ancestry and K10 to African (AFR) ancestry. Furthermore, Indigenous groups (K3–K6) can be further separated by PC3 (Supplementary Fig. 5b and Supplementary Data 3).

We investigated the coancestry patterns and fine-scale population structure, focusing on admixed subgroups K1–K6 (see Methods, ‘Population structure analysis’). The resulting coancestry heat map reveals distinct blocks of haplotype sharing consistent with population differentiation (Fig. 3b). Two major clusters, corresponding to Han Chinese-enriched (K1 and K2) and Indigenous-enriched (K3–K6), are clearly observed, with limited haplotype sharing between the clusters, indicating substantial genetic divergence.

Within the Han Chinese-enriched cluster, subgroups K1 and K2 form a tightly linked block, characterized by high intra-cluster haplotype sharing (pink to blue shading in Fig. 3b), which reflects recent common ancestry and low differentiation. By contrast, the Indigenous-enriched cluster shows greater internal heterogeneity. Subgroups K3 and K6 share a moderate level of haplotypes, whereas K4 and K5 exhibit lower within-group sharing and form more isolated sub-blocks, suggesting stronger drift or founder effects. Notably, K4 and K5 show the lowest haplotype sharing with Han Chinese (yellow and orange in the corresponding cells), supporting a longer-term separation or minimal historical gene flow.

The hierarchical clustering dendrogram further supports this differentiation: Han populations are grouped separately from Indigenous clusters, and substructure among Indigenous groups reflects differing levels of drift and gene flow. These results are consistent with a model in which Han Chinese and Indigenous Taiwanese groups have experienced distinct demographic trajectories, with Indigenous subgroups exhibiting substantial population-specific drift and complex internal differentiation, potentially shaped by geographical isolation, cultural boundaries and ancient settlement patterns.

We reconstructed the demographic relationships and detected admixture from K1 to K6. The resulting best-fit graph (score = 0.5408) reveals a bifurcated structure in which K1 and K2 cluster tightly into a Han-specific lineage characterized by short internal drift edges, indicating recent common ancestry and limited genetic differentiation. By contrast, K3–K6, with a smaller population size compared with the Han Chinese, trace their ancestry to more basal lineages with longer edge lengths (for example, K5 and K6), consistent with early divergence and population-specific drift after isolation. Overall, these results support clear differentiation between Han and Indigenous populations, and they also identify recent and ancient signals of admixture (Fig. 3c).

In addition, homozygosity analysis (see Methods, ‘Homozygosity analysis’) reveals that individuals who are closer to the Indigenous and non-East Asian ethnic groups exhibit higher homozygosity (Supplementary Fig. 7a and Supplementary Data 7), and that cohorts with East Asian ancestry (TPMI, TWB and EAS) show lower homozygosity, compared with other groups of non-East Asian ancestry (Supplementary Fig. 7b and Supplementary Data 8). An explanation of these results is provided in Supplementary Note 5.

Our analysis of population genetic structure, with a large sample size and a complete representation of Taiwanese populations—including individuals of Han Chinese ancestry, recognized Indigenous groups, unrecognized Plains Indigenous ancestry groups (Pingpu), global immigrants, and their admixture—provides detailed insight into the genetic admixture of the Taiwanese population.

## GWASs, QTL mapping and sample size

As regards sample-size evaluation (see Methods, ‘Evaluation of sample size’), to detect a SNP that has an odds ratio from 1.1 to 2.0 for a condition based on a MAF of 0.01–0.25, case/control ratio of 1:4 and significance level of 5 × 10^−8^, the sample size required for attaining a statistical power of 0.8 was calculated by QUANTO^26^ (Supplementary Fig. 8a and Supplementary Data 9). To detect a quantitative trait locus (QTL) that has a beta coefficient from 0.02 to 0.20 for a condition based on a MAF of 0.1–0.25, and a significance level of 5 × 10^−8^, the sample size required for attaining a statistical power of 0.8 was calculated by QUANTO (Supplementary Fig. 8b and Supplementary Data 10). For example, after rigorous data quality control (see Methods, ‘Evaluation of DNA contamination’ and ‘Quality control’; Supplementary Figs. 9 and 10), our GWASs (see Methods, ‘GWASs, QTLs and functional annotation’) for T2D (*n* = 52,290 cases and 192,817 controls; Supplementary Fig. 8c, top and Supplementary Data 11), haemoglobin A1c (HbA1c; *n* = 140,259; Supplementary Fig. 8c, bottom and Supplementary Data 11), EHT (*n* = 71,548 cases and 130,561 controls; Supplementary Fig. 11a and Supplementary Data 12), systolic blood pressure (SBP; *n* = 241,667; Supplementary Fig. 11b and Supplementary Data 13) and diastolic blood pressure (DBP;* n*  = 241,646; Supplementary Fig. 11c and Supplementary Data 14) replicate previous findings (Supplementary Note 6). Sensitive analyses that consider a higher SBP threshold for EHT obtained reasonably consistent results across the three SBP cut-offs (Supplementary Figs. 11a and 12a,b and Supplementary Data 12, 15 and 16), illustrating the robustness of the GWASs.

**Fig. 4:**
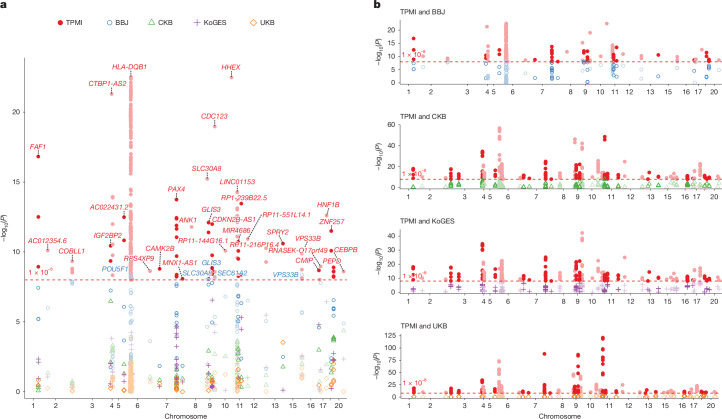
Comparison of T2D GWAS results in the TPMI with those from four biobanks. **a**, Comparison of GWAS results from the TPMI and the PheWebs of the Biobank Japan (BBJ), China Kadoorie Biobank (CKB), the Korean Genome and Epidemiology Study (KoGES) and the UK Biobank (UKB). A Firth logistic regression was applied for the T2D GWAS in the TPMI. All statistical tests were two-sided. Multiple-testing adjustment was applied using a genome-wide significance threshold of *P* < 1 × 10^−8^. Novel T2D-associated SNPs identified in our GWAS but absent in biobanks at the genome-wide significance level are shown. **b**, Pairwise comparison with each biobank. Different statistical methods were applied across cohorts. For BBJ, CKB and KoGES, a generalized linear mixed model was implemented using SAIGE; for UKB: linear regression was used. All tests were two-sided. Bonferroni correction was applied for multiple-testing adjustment across loci. The four graphs (from top to bottom) show T2D-associated SNPs identified in the TPMI but not in BBJ, CKB, KoGES and UKB, respectively. Source Data.

The results of our GWASs for T2D, HbA1c, EHT, SBP and DBP were compared with those from the PheWebs of the Biobank Japan (BBJ)^27^, China Kadoorie Biobank (CKB)^28^, Korean Genome and Epidemiology Study (KoGES)^29^ and UK Biobank (UKB) (Fig. 4 and Supplementary Data 17). Replicable association signals were identified across populations of East Asian and European ancestry (Supplementary Data 17). In addition, novel T2D-associated SNPs uniquely identified in our GWASs, but absent in biobanks at the genome-wide significance level, are shown. For example, our GWAS for T2D identified dozens of novel T2D-associated SNPs in genes such as *HLA-DQB1*, *C2*, *VWA7*, *MSH5-SAPCD1*, *LY6G5B*, *CLIC1*, *LY6G6C*, *BTNL2*, *EHMT2*, *NELFE*, *HCG23*, *SLC44A4*, *ATP6V1G2*, *HLA-DOA*, *TNXB*, *MUC22*, *SKIV2L*, *PSORS1C1*, *CDSN*, *DXO*, *PSORS1C2*, *TCF19*, *CCHCR1*, *POU5F1*, *HCG22*, *BRD2*, *IP6K3*, *LINC01016*, *CDKN2B-AS1*, *LOC100420530* and *SMG6*, among others (Supplementary Data 17), highlighting that the TPMI, with its large sample size, provides new and powerful resources for gene mapping and precision medicine. Notably, we also found that different GWASs identified some different SNPs within the same gene (Supplementary Data 17), reflecting allelic heterogeneity, variation in linkage disequilibrium patterns and differences in genetic background across populations with East Asian ancestry. For detailed citation information for replicating previous findings in the GWAS and QTL results, see Supplementary Data 18. Functional annotation of the identified novel T2D-associated SNPs, done using ANNOVAR^30^ (see Methods, ‘GWASs, QTLs and functional annotation’), is provided in Supplementary Data 19. Furthermore, disease and bio-function analysis of the genes associated with these novel SNPs revealed enrichment in diabetes (*P* = 3.18 × 10^−08^–7.77 × 10^−11^), cardiovascular diseases (*P* = 1.55 × 10^−18^–2.71 × 10^−20^), metabolic diseases (*P* = 2.65 × 10^−10^) and inflammatory diseases (*P* = 4.59 × 10^−07^–3.99×10^−09^), which are biologically relevant to T2D (Supplementary Data 20). These findings highlight the potential genetic correlations between T2D and its comorbidities in the TPMI cohort, demonstrating the value of large, population-specific datasets in uncovering novel, functionally relevant variants.

Additional results from GWASs and phenome-wide association studies (PheWASs) for important diseases in Taiwan and their subtypes, as well as quantitative traits, are reported in another study^31^. Generalized mixed-effect analysis using REGENIE^17^ also yields similar results (Supplementary Fig. 13a–e and Supplementary Data 21–25). These results show that the TMPI cohort can be used to uncover the genetic underpinnings of complex disorders and traits such as T2D and EHT.

## PRSs

**Fig. 5:**
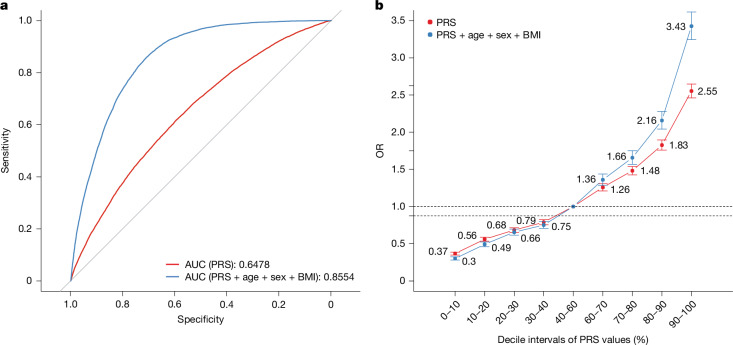
PRS analysis for T2D. **a**, AUC of PRS (red curve) and of PRS, age, sex and BMI (blue curve). **b**, Dose–response effect of PRS levels on the odds ratio (OR) of T2D. Dose–response effect of PRS (red line) and of a combination of PRS, age, sex and BMI (blue curve) with *n* = 205,779 independent samples. Error bars represent the 95% confidence interval, calculated as exp (*β* ± *Z*_0.025_ × s.e.), based on maximum likelihood estimation from a logistic regression model with different decile intervals of PRS values included as covariates. The estimated coefficient (*β*) is provided in column B (‘Estimate’) and the standard error (s.e.) is provided in column C (‘Std. Error’) in the Source Data for **b**. Source Data.

In the example of T2D, two multi-ancestry PRSs were constructed (see Methods, ‘PRSs’). The first method, which applied the summary genetic effects from meta-GWASs comprising one million participants with East Asian, European and South Asian ancestry in the DIAGRAM Consortium^32^, resulted in an area under the receiver operating characteristic curve (AUC) of 0.65 in both the training and the testing datasets. After incorporating age, sex and body mass index (BMI), the AUC increased further to 0.86 in both the training and testing datasets (Fig. 5a). The second PRS, based on the effect sizes in polygenic score (PGS) (PGS002308)^33^, yielded a similar AUC to that of the first PRS (Supplementary Fig. 14a and Supplementary Data 26). The positive dose–response correlation between the PRS level and the T2D odds ratio (Fig. 5b, Supplementary Fig. 14b and Supplementary Data 27) was enhanced after incorporating demographic factors such as age, sex and BMI (Fig. 5a, Supplementary Fig. 14a and Supplementary Data 26), highlighting the potential of PRSs for identifying individuals who have a heightened risk of T2D, and thus enabling targeted interventions and more precise therapeutic strategies. The TPMI has developed PRSs for 265 dichotomized phecodes and 24 quantitative traits, covering major common diseases in Taiwan^31^. These PRSs will be instrumental for developing models for disease risk assessment, and for advancing the integration of artificial intelligence into precision medicine, particularly for high-incidence diseases.

## Discussion

The TPMI has reached a cohort size of more than 500,000 participants of Han Chinese ancestry, with genetic and EMR data available for analysis. The genetic homogeneity and richness of clinical data (from years before enrolment, together with those from future hospital visits) make this large cohort of individuals with non-European ancestry highly valuable for genetic and epidemiological research. As reported elsewhere, results from large-scale GWASs and studies of common disease risk prediction (based on PRS)^31^, deleterious variants^34^ and pharmacogenetics^35^ in the TPMI cohort have real-world implications. The commitment to return study results to the participants and involve them in future clinical research will facilitate the validation of precision medicine approaches in healthcare management.

However, the TPMI cohort has two sets of limitations. First, the quantity and quality of the clinical data are not perfect. Although the TPMI participants grant us access to ‘all’ of their clinical data, the project lacks the resources and workforce to retrieve hospital data that are in archival storage, which means that our access to data that were collected before the participants enrolled in the project is limited. In addition, because many patients receive care from multiple hospitals and clinics under Taiwan’s National Health Insurance Program, clinical data from sources outside of the hospital through which a participant enters the project are unavailable to us. These circumstances result in incomplete clinical data, leading to cases in which the age of disease onset, test results, treatments prescribed and drug responses are missing. In addition, the TPMI did not collect all EMR variables in a standardized manner at the same time, so baseline time and data vary by participant. Second, owing to technical constraints, the genetic risk profile data do not encompass some known risk variants. Although many known risk variants are included in the SNP array, some cannot be genotyped because suitable probes cannot be designed on the array. Furthermore, the genotyping accuracy of SNP arrays is low when the MAF of the marker is lower than 0.1%; this makes it challenging to confidently type a substantial fraction of the known risk variants in the TPMI cohort, because they are scarce.

De-identification of EMRs is crucial for privacy protection. The primary EMR format used in the TPMI follows the HL7 CDA R2 standard, as defined by Taiwan’s EMR Exchange Center. The format includes various report types that may contain free-text data, such as image reports, operation notes, pathology reports, discharge summaries and family histories, collectively constituting approximately 4% of the medical record. Hospitals remove personal identifiers from structured data to protect participant privacy before participating in the EMR exchange. Free-text data are subjected to a multi-stage de-identification process using pattern-matching algorithms to eliminate sensitive information, including phone numbers, email addresses, multiple types of ID, birth dates and addresses. Removing Chinese names is particularly challenging, and is handled through the following two pipelines: (1) pattern matching: identifying and removing names from specific sections of certain report types; (2) natural language processing (NLP): using the CKIP NLP toolkit to detect and eliminate personal names in a more dynamic manner. These two pipelines are applied to each record, serving as cross-validation mechanisms to enhance accuracy. The de-identification pipelines are regularly updated to accommodate new EMR formats and hospital-specific variations. Despite the robustness of this approach, full-text de-identification remains a complex task, which requires ongoing refinement and rigorous human validation to ensure comprehensive coverage and accuracy.

### Future directions

The main focus of the TPMI is to develop algorithms to predict disease risk for as many conditions as the cohort can support. Once developed, the algorithms must be validated in real-world settings before being adopted for the population. The TPMI cohort will be very useful in this regard. For common diseases that affect older people, there will be some individuals in the TPMI cohort who are in the high-risk group for each disease but are not yet affected. Following these individuals, especially those approaching the expected age of onset, can provide a measure of the predictive power of the algorithms. Furthermore, for some high-risk groups for which health management strategies are available for the diseases in question, a trial comparing those who follow the risk-lowering guidelines versus those who are treated with the standard of care will determine whether genetic-risk-guided health management is beneficial. For example, those with a high risk of cancer could be enrolled in an early screening programme, and those with high stroke risk could be enrolled in a stroke prevention programme that includes blood pressure control and smoking cessation. To make the cohort even more helpful, additional resources will be sought to retrieve archival clinical data from the hospitals through which the participants join the TPMI and to obtain consent from the participants to extract data from the National Health Insurance Database^36,37^ and other hospitals and clinics in which they receive care. With this enhanced dataset and longitudinal follow-up data, the TPMI cohort can be studied for years to come.

In addition to the TPMI, TWB^19,20^ and China Medical University Hospital (CMUH)^38^ represent two additional large cohorts for genetic studies in Taiwan. TWB and CMUH have recruited 200,000 and 170,000 participants, respectively. TWB aimed for broad recruitment across Taiwan, whereas CMUH focused more regionally. The integration of TPMI, TWB and CMUH data forms one of the largest cohorts for genetic studies globally, significantly enhancing statistical power for GWASs and PRSs in the East Asian population. However, this integration also presents challenges for genetic analysis, owing to differences in study design and data collection among the three large cohorts. For instance, TWB collected self-reported disease records through questionnaires rather than through medical diagnosis in EMRs, and CMUH focuses mainly on patients from central Taiwan. These differences among the three cohorts produce difficulties for data analysis. CKB^39^ and Precision Health Research, Singapore (PRECISE)^40^ are two other large genetic resources that include a considerable number of participants with Han Chinese ancestry. A meta-analysis based on summary statistics might offer a viable approach, but careful adjustment for potential confounders and background differences among the cohorts is necessary, and advanced methods should be developed.

Return of Results (RoR) is crucial to empower participants through engagement and education, raise public awareness of precision health and facilitate the establishment of infrastructure for clinical implementation. For the TPMI project, an RoR platform for 83 genetic conditions has been developed with custom-designed RoR web pages by each hospital. The content includes disease-related variants and pharmacogenetics-related variants. For disease-related variants, founder mutations or pathogenic variants with multiple evidence (following the guidelines of the American College of Medical Genetics and Genomics (ACMG) and the National Comprehensive Cancer Network (NCCN)) related to cancer, dermatology, endocrine, hearing loss, haematology, metabolism, neurology and ophthalmology are interpreted (Supplementary Table 6). For pharmacogenetics-related variants, actionable variants from the US Food and Drug Administration (FDA) and Clinical Pharmacogenetics Implementation Consortium (CPIC) are selected, and the therapeutic ranges of the drugs involved include anaesthesiology, anti-inflammatory, cardiology, endocrinology, gastroenterology, haematology, hyperuricaemia, infectious diseases, muscle tenderness, neurology, oncology, psychiatry, toxicology and transplantation (Supplementary Table 6).

Genetic consultation is essential in guiding individuals, families and society through life-changing decisions based on genetic information, especially in implementing ROR. Prioritizing genetic consultation within the TPMI will address several key considerations, including facilitating informed decision-making, safeguarding the privacy and confidentiality of genetic data, navigating ethical complexities, providing psychosocial support for emotional challenges and ensuring equitable access to precision health services across diverse demographic populations. The TPMI is positioned to transform healthcare by blending scientific progress with ethical awareness, particularly through advancing precision health. Recognizing the crucial role of genetic consultation in managing ethical, legal, and social implications (ELSI) in Taiwan, the TPMI will be dedicated to harnessing the power of genetic data while valuing the guidance provided by genetic counsellors in navigating ELSI complexities.

## Methods

### Genotyping and plate normalization

Genomic DNA was purified automatically from 200 μl whole blood with the QIAsymphony DSP DNA Mini Kit (QIAGEN), and 15 μl of genomic DNA at 50 ng μl^−1^ was subjected to genotyping using Axiom TPMv1 (Axiom TPM) or TPMv2 Array (Axiom TPM2) (Thermo Fisher Scientific) according to the manufacturer’s instructions. Genotyping assays were performed at the National Center for Genome Medicine at the Academia Sinica, Taipei, Taiwan (https://ncgm.sinica.edu.tw/) and six partner hospitals, including the Center of Applied Genomics at Kaohsiung Medical University, Kaohsiung, Taiwan; the Precision Medicine Center at Taichung Veterans General Hospital, Taichung, Taiwan; Chang Gung Memorial Hospital, New Taipei City, Taiwan; Chuanghua Christian Hospital, Chuanghua, Taiwan; Hualien Tzu Chi Hospital, Hualien, Taiwan; and Taipei Medical University, Taipei, Taiwan. Genotype calling was performed for approximately 3,000 individuals (ranging from 2,304 to 3,936) per batch using Applied Biosystems Array Power Tools (APT) as part of the Best Practices Workflow at the National Center for Genome Medicine at the Academia Sinica. Each batch included arrays from consecutive assays at individual centres to minimize the potential batch effect. Individuals with a call rate of 98% or lower were genotyped again to improve the call rate.

We also performed plate normalization to examine whether there were differences in the signal distribution of 96-well plates within the same calling batch for each marker. Variations in signal intensity across different plates can lead to misjudgement by the clustering algorithm during the genotype calling stage. The normalization procedure helps to mitigate such issues, reduce the effect of signal intensity variations and improve the clustering accuracy. We calculated the allele frequency of each marker to identify any abnormalities and, from these calculations, determined whether normalization was required.

### Imputation

Whole-genome sequencing data using Illumina HiSeq and Novaseq from 1,498 individuals from the TWB were used as imputation references^25^. The sequencing reads were aligned to the human genome reference GRCh38 using BWA^41^. Variants were called jointly with DeepVariant^42^. Read-based phasing was done with WhatsHap^43^ at first, followed by population-level phasing with SHAPEIT4 for better accuracy^44^. Removal of variants with a minor allele count lower than 2, a Hardy–Weinberg equilibrium test *P* value of less than 1 × 10^−10^ or a missing rate greater than 5% resulted in 22.44 million genetic variants in the imputation reference panel. SHAPEIT5 and IMPUTE5 were applied to all genotyped individuals for haplotype phasing and genome imputation^45,46^.

### Familial relatedness analysis

For the samples that passed sex, inconsistent duplicated EMR and call-rate checks, we used a dataset comprising 485,925 individuals and 68,741 unlinked SNPs to estimate familial relationships using KING software (v.2.2.7)^47^. These SNPs were selected according to the following criteria: MAF greater than 5%; SNP call rate of at least 99%; and pairwise linkage disequilibrium of less than 0.3 within a sliding window of 5 Mb. Inference of close relationships, such as duplicates or monozygotic twins (Dup/MZ), parent–offspring (PO), full siblings (FS), second-degree (2nd) and third-degree (3rd), was done using the ‘-related’ option, which estimates kinship coefficient by the proportion of genomes shared identical by descent.

### Population structure analysis

The population structure of the TPMI cohort was assessed against external resources with known population information from various genetic projects, including the TWB^19,20^, the SGDP^21^ and the 1KGP^22^. The TWB dataset encompassed 83,664 individuals, consisting of 68,023 with Minnan ancestry, 11,549 with Hakka ancestry and 4,092 Han Mainlanders, further categorized into 1,681 Southern Han, 1,606 Central Han and 805 Northern Han on the basis of self-reported birth geographical regions. The SGDP dataset included three individuals from two Taiwan Indigenous tribes—namely, one Atayal individual and two Ami individuals—to assess the genetic contribution of Indigenous populations in Taiwan. Within the 1KGP dataset, there were 3,202 individuals representing 26 global populations across 5 continents: Africa (AFR), the Americas (AMR), East Asia (EAS), South Asia (SAS) and Europe (EUR). This dataset comprised 893 individuals with AFR ancestry, 490 with AMR ancestry, 585 with EAS ancestry, 601 with SAS ancestry and 633 with EUR ancestry. The EAS-ancestry group consisted of 104 Japanese in Tokyo, Japan (JPT), 103 Han Chinese in Beijing, China (CHB), 163 Southern Han Chinese, China (CHS), 93 Chinese Dai in Xishuangbanna, China (CDX) and 122 Kinh in Ho Chi Minh City, Vietnam (KHV).

PCA was performed based on a set of 234,255 autosomal SNPs common to both TPMv1 and TPMv2 SNP arrays. These SNPs passed stringent quality-control measures, including an MAF greater than 1% and a call rate exceeding 99%. In addition, SNPs with an inter-marker linkage disequilibrium of *r*^2^ less than 0.2 were chosen. The analysis comprised 70,708 TPMI individuals who passed stringent sample quality-control criteria and were born before 1950, referred to as ‘<1950’. The first two PCs, which explained 43.9% and 18.8% of the genetic variation, respectively, were derived from the analysis involving a total of ten PCs, as calculated from their corresponding eigenvalues. These components were used to construct a reference coordinate system. Subsequently, all other participants, including the individuals of ‘>1950’ in the TPMI, TWB and SGDP, were projected onto this reference coordinate system. For computational efficiency, PCA and the top 10 PCs were generated using the fastPCA version (--pca approx) in PLINK 2.0.

Genetic ancestry fractions were estimated using ADMIXTURE (v.1.3.0)^48^. To ensure adequate representation of diverse genetic backgrounds in model building, particularly for samples that showed distinct genetic patterns in the PCA plot, we first divided the TPMI samples into nine equal quadrants according to their PC1 and PC2 coordinates. Random sampling was performed within each quadrant to create a representative dataset of 33,778 individuals for model building. The optimal number of ancestral populations (*K*) was determined using ADMIXTURE’s cross-validation procedure, evaluating values from *K* = 5 to *K* = 18, with *K* = 10 showing the lowest cross-validation error (Supplementary Fig. 6a and Supplementary Data 4). To further assess the robustness of this choice, we examined the stabilization pattern of incremental improvements in log-likelihood across different *K* values (Supplementary Fig. 6b and Supplementary Data 5) and quantified genetic differentiation among inferred subgroups (admixed populations) at the optimal *K* using the pairwise genetic distance (*F*_ST_) using ADMIXTURE. A hierarchical clustering dendrogram based on average linkage of the *F*_ST_ matrix (Supplementary Table 5) was then constructed to visualize the genetic relationships among the ten inferred admixed subgroups (Supplementary Fig. 6c and Supplementary Data 6). Finally, the resulting ancestry model was used to project the remaining TPMI samples for estimating ancestry admixture fractions.

To investigate coancestry and fine-scale population structure, we selected a representative subset of 600 individuals, comprising 100 individuals from each of 6 subgroups (K1–K6) identified in our previous ADMIXTURE analysis. These subgroups included Han Chinese (K1 and K2) and four Indigenous populations (K3–K6), with individuals chosen on the basis of high admixture proportions representative of their respective groups. Genotype data on chromosome 6 were phased using SHAPEIT (v.2, release 900)^49^ to obtain haplotype information. The phased data were then converted into the required PHASE and RECOMBFILES formats using utility scripts provided with ChromoPainter^50^. Haplotype sharing and coancestry were inferred using ChromoPainter, which models each individual’s genome as a mosaic of haplotypes copied from others, enabling the quantification of haplotype donation between individuals. The resulting coancestry matrix, which reflects the proportion of the genome each shares with others, was subsequently analysed using fineSTRUCTURE (v.4)^50^ to perform Bayesian clustering and infer fine-scale population structure and individual relationships on the basis of shared ancestry patterns.

To infer population admixture patterns and reconstruct demographic history, we applied ADMIXTOOLS 2^51^ to the same dataset of 600 individuals used in our coancestry analysis. We used the find_graphs() function to systematically explore plausible admixture graph topologies, allowing up to three admixture events per model. This automated procedure evaluates candidate models on the basis of their fit to empirical *f *statistics, including *f*_2_, *f*_3_ and *f*_4_, which capture genetic drift, shared ancestry and admixture signals, respectively. Each candidate graph was subsequently refined using the qpgraph() function, which optimized drift edge lengths and admixture proportions by minimizing the negative log-likelihood, calculated as the squared difference between observed and fitted *f*_2_ statistics. To improve convergence and mitigate the risk of local optima, we implemented an iterative optimization strategy over 1,000 iterations, using a different random seed for each run. In each iteration, the best-fitting graph (the one with the lowest likelihood score) was used as the initial graph for the subsequent round via the initgraph parameter. Among all models evaluated, the admixture graph that achieved the lowest likelihood score was selected as the optimal model, representing the best overall fit to the observed data.

### Homozygosity analysis

The homozygosity rate of each individual was calculated using PLINK 2.0 (--het), based on 479,610 autosomal SNPs shared in TPMv1 and TPMv2. To observe a homozygosity pattern in the TPMI, a smoothing homozygosity rate was calculated as follows. PCA based on genotype data (Supplementary Fig. 7a and Supplementary Data 7) was performed, and the coordination of the first two PCs was divided into 150 evenly spaced partitions. The partitions that contained no individuals were removed, resulting in 4,739 partitions remaining. In each PC partition, homozygosity rates of individuals were calculated and visualized in a heat map (Supplementary Fig. 7a and Supplementary Data 7). In addition, cross-ancestry distributions of individual’s whole-genome homozygosity rates were visualized in violin plots for comparison (Supplementary Fig. 7b and Supplementary Data 8).

### Evaluation of sample size

We used QUANTO 1.2.4 (quantitative trait loci analysis tool)^26^ to calculate the required sample sizes for identifying a disease-associated SNP in a GWAS or a QTL in a QTL mapping to attain a statistical power of 0.8 under a genome-wide significance level of 5 × 10^−8^ for various situations. For binary traits, on the basis of a logistic regression, we considered SNPs with an MAF ranging from 0.01 to 0.25 and an odds ratio ranging from 1.1 to 2.0, under the ratio of cases to controls of 1 to 4 (Supplementary Fig. 8a and Supplementary Data 9). For quantitative traits, on the basis of a linear regression, we considered SNPs with an MAF ranging from 0.01 to 0.25 and an effect size ranging from 0.02 to 0.2 (Supplementary Fig. 8b and Supplementary Data 10).

### Evaluation of DNA contamination

For the genotyping technology used in this study, DNA contamination is indicated by a high dish quality control (DQC) accompanied by a low quality control call rate (QCCR) and final call rate. In addition, if a DNA sample is contaminated by another sample from an individual of a different gender, the estimated gender based on probes on the XY chromosomes will be unknown. An expert manually examined the genotype data to exclude samples showing signs of DNA contamination. Blood DNA was recollected from the same individuals, and a genotyping assay was performed again. Through this procedure, the number of samples with DNA contamination included in the dataset was minimized.

### Quality control

We performed sample and SNP quality control using PLINK in combination with KING and R (ref. ^52^; Supplementary Figs. 9 and 10). At first, there were 486,956 participants with both EMR and genotyping data in either the TPMv1 array (*n* = 165,956) or the TPMv2 array (*n* = 321,360), and 479,610 shared autosomal SNPs genotyped on both arrays. We began by excluding SNPs in specific batches of participants with significantly different allele frequencies compared with other batches. Subsequently, we sequentially identified and removed 705 participants with inconsistencies between EMR-recorded gender and genetic gender determined by homozygosity pattern of the X chromosome; 307 participants, assembled into 2-to 3-person groups by highly similar genomes, who had inconsistent EMR records for either genders or birth dates; 19 participants with a low genotyping call rate (GCR) of less than 0.95 (--mind 0.05); 8,123 participants with an autosomal heterozygosity rate (--het) more than three standard deviations away from the mean of heterozygosity rates; 111,489 participants with equal to or higher than 2nd-degree cryptic relations with other participants estimated by KING; and 1,135 participants who deviated from 99.99% confidence bands (R {car}) of the first two PCs of genetic relationship matrix projected onto the 1KGP dataset (--score). Here, we retained 365,178 independent participants (independent-samples dataset) for the subsequent GWAS and PRS analyses. For GWAS analyses based on a mixed-effect model, which allows samples to be related, 476,449 related participants (related-samples dataset) were included for the subsequent GWAS analyses. On the basis of unrelated participants, we hierarchically excluded SNPs for each studied trait by GCR of less than 0.95 (--geno 0.05) or those that failed the nonrandom missingness test for a binary trait (--test-missing), MAF of less than 0.01 (--maf 0.01) and *P* value of the Hardy–Weinberg equilibrium test at the Bonferroni’s level (--hwe). Finally, approximately 440,000 filtered SNPs remained for each trait.

### GWASs, QTLs and functional annotation

We performed GWASs for two binary disease traits: T2D and EHT. We also performed QTL mappings for three quantitative traits: glycated haemoglobin (HbA1c), systolic blood pressure (SBP) and diastolic blood pressure (DBP).

For binary disease traits, we defined disease status using ICD-10 codes from EMR records with laboratory tests. A patient with T2D was defined as having at least ten records of ICD-10 code E11 or having HbA1c ≥ 6.5% and fasting glucose (FS) ≥ 126 mg dl^−1^. A non-T2D control was defined as having none of the ICD-10 codes related to diabetes mellitus (DM) and none of the records with HbA1c over 5.6% or FS over 100 mg dl^−1^. Similarly, a patient with EHT was defined by the ICD-10-code I10, SBP ≥ 120 mmHg, or DBP ≥ 80 mmHg. A control individual without EHT (non-EHT control) did not meet any of the aforementioned EHT inclusion criteria. In addition, sensitivity analyses were performed considering different SBP cut-offs (SBP ≥ 130 or SBP ≥ 140 mmHg) for defining EHT in a GWAS.

Firth logistic regression with age, sex, BMI and ten PCs for ancestry adjustments was implemented by PLINK 2.0 (--glm) on an independent-samples dataset. In addition, a logistic mixed-effect model adjusted by the same covariates (age, sex, BMI and ten PCs) was implemented by REGENIE (v.4.1) on a related-samples dataset. For quantitative traits, we first applied the inverse normal transformation^53^ to the residuals obtained by regressing the quantitative trait against the aforementioned covariates. Subsequently, a linear regression was implemented by PLINK on an independent-samples dataset, and a linear mixed-effect model was implemented by REGENIE on a related-samples dataset.

Detailed functional annotation of the novel SNPs associated with T2D, EHT, HbA1c, SBP and DBP was done using ANNOVAR (release: 2020-06-08)^30^ with the table_annovar.pl script. To further investigate the biological relevance of these variants, gene set enrichment and pathway analyses were performed using ingenuity pathway analysis (IPA)^54^, using curated data from the Ingenuity Knowledge Base to identify enriched canonical pathways and biological functions associated with SNP-linked genes.

### PRSs

We computed multi-ancestry PRSs for T2D using a Bayesian approach, PRS-CSx^55^, which integrates the TPMI imputation data and the summary genetic effects from meta-GWASs that were done in various ethnic populations in DIAGRAM^32^ through a shared continuous shrinkage prior. The population ancestries include: (a) East Asian ancestry: 283,423 individuals (56,268 cases and 227,155 controls); (b) European ancestry: 933,970 Caucasian individuals (80,154 cases and 853,816 controls); and (c) South Asian ancestry: 49,492 individuals (16,540 cases and 32,952 controls). Moreover, ancestry-matched linkage disequilibrium references were extracted from the EAS, EUR and AFR groups in the 1KGP^22^; 9,106,250, 10,454,875 and 10,401,621 SNPs for EAS, EUR and SAS were merged with the TPMI imputation data to calculate the population-specific PRS for each individual using the PLINK (--score command). SNP effect sizes across the three population-specific PRSs were combined using an inverse-variance-weighted meta-analysis of population-specific posterior effect size estimates to calculate a final PRS (--meta command).

In addition, we applied the PGS for T2D (PGS002308) from the PGS Catalog^33^, in which the SNP effect sizes were estimated from 23,827 individuals with African American ancestry, 177,415 individuals with East Asian ancestry and 898,130 individuals with European ancestry using PRS-CSx^56^.

The TPMI participants were divided into 205,779 independent and 62,304 related participants. Logistic regression models, with T2D disease status as a dichotomous response variable and PRS with and without demographic variables (age and sex) and BMI as independent variables, were established on the basis of the independent participants. Finally, the models were applied to the related participants to evaluate the model performance, assessed by AUC.

### Ethics statement

This study was approved by the Institutional Review Boards of Taipei Veterans General Hospital (2020-08-014 A), National Taiwan University Hospital (201912110RINC), Tri-Service General Hospital (2-108-05-038), Chang Gung Memorial Hospital (201901731A3), Taipei Medical University Healthcare System (N202001037), Chung Shan Medical University Hospital (CS19035), Taichung Veterans General Hospital (SF19153A), Changhua Christian Hospital (190713), Kaohsiung Medical University Chung-Ho Memorial Hospital (KMUHIRB-SV(II)-20190059), Hualien Tzu Chi Hospital (IRB108-123-A), Far Eastern Memorial Hospital (110073-F), Ditmanson Medical Foundation Chia-Yi Christian Hospital (IRB2021128), Taipei City Hospital (TCHIRB-10912016) and Koo Foundation Sun Yat-Sen Cancer Center (20190823A), Cathay General Hospital (CGH-P110041), Fu Jen Catholic University Hospital (FJUH109001) and Academia Sinica (AS-IRB01-18079), Taiwan. Written informed consent was obtained from the participants in accordance with institutional requirements and the Declaration of Helsinki principles. All collected information was de-identified before statistical data analysis.

### Reporting summary

Further information on research design is available in the Nature Portfolio Reporting Summary linked to this article.

## Online content

Any methods, additional references, Nature Portfolio reporting summaries, source data, extended data, supplementary information, acknowledgements, peer review information; details of author contributions and competing interests; and statements of data and code availability are available at 10.1038/s41586-025-09680-x.

## Supplementary information

Supplementary Information Supplementary Notes 1–6, Supplementary Figs. 1–14, Supplementary Tables 1–6, descriptions for Supplementary Data 1–27 (data supplied separately) and Supplementary References.

Reporting Summary

Supplementary Data 1–13 See Supplementary Information for descriptions.

Supplementary Data 14–20 See Supplementary Information for descriptions.

Supplementary Data 21–23 See Supplementary Information for descriptions.

Supplementary Data 24–27 See Supplementary Information for descriptions.

## Source data

Source Data Figs. 1, 2, 4 and 5

## Data Availability

All summary statistics and results from this study are freely available from the TPMI website (https://tpmi.ibms.sinica.edu.tw). In compliance with the data protection laws governing genetic and health data in Taiwan, the de-identified TPMI clinical and genotyping data are kept in a secure server at the Academia Sinica and not released to the public. The TPMI is in the process of transitioning to a biobank model, which will make the TPMI data openly accessible to the public for research purposes by December 2026, and data analysis will be performed on local servers in a trusted research environment. Researchers who wish to access the individual clinical and genotyping data before that time can do so by collaboration through a four-step process. (1) Complete an online application form posted on the TPMI website with specific proposals for collaboration. (2) The TPMI Feasibility Committee will assess the scientific, clinical, technical, resource, and regulatory feasibility of the proposals at a monthly meeting and approve all feasible proposals, with priority given to those aligned with TPMI’s mission, resource capacity and compliance obligations. (3) A TPMI team will work with the applicant of the approved proposal to prepare a protocol of the project for review by the Academia Sinica Institutional Review Board (IRB). (4) Once the IRB approval is obtained, the collaborative work will be performed by the TPMI team according to the collaborators’ proposed study design. Summary statistics and analysis results will be delivered to the collaborators. In addition to the TPMI data, we analysed the following external datasets as part of the validation: - Meta-GWAS summary statistics for T2D across multiple populations from the DIAGRAM Consortium are available at https://diagram-consortium.org/downloads.html. - The linkage disequilibrium reference from various populations of the 1KGP can be downloaded from https://github.com/getian107/PRScsx. - PRS-CSx weights for T2D across multiple populations from the PGS Catalog are available at https://www.pgscatalog.org/score/PGS002308/. - The 1KGP data can be accessed through PLINK 2.0 at https://www.cog-genomics.org/plink/2.0/resources#phase3_1kg. - Fastq files of SGDP samples are available at https://www.internationalgenome.org/data-portal/data-collection/sgdp. - Genotype data from the TWB are available through a formal application process (https://www.twbiobank.org.tw/index.php). - Biobank Japan (BBJ) GWAS summary statistics can be assessed through the BioBank Japan PheWeb (https://pheweb.jp). - China Kadoorie Biobank (CKB) GWAS summary statistics can be assessed through the China Kadoorie Biobank PheWeb (https://pheweb.ckbiobank.org). - Korean Genome and Epidemiology Study (KoGES) GWAS summary statistics can be assessed through the KoGES PheWeb (https://koges.leelabsg.org). - UK Biobank (UKB) GWAS summary statistics can be assessed through the UK Biobank PheWeb (https://pheweb.org/UKB-Neale). In addition, to support researchers in data exploration, TPMI has developed several platforms, as follows. (1) TPMI PheWeb (https://pheweb.ibms.sinica.edu.tw), a user-friendly interface that allows researchers to explore associations between genetic variants and phenotypes. It provides access to summary statistics from GWASs across a broad range of phenotypes and traits. (2) TPMI SNPView (https://tdap.ibms.sinica.edu.tw/snpview/), a comprehensive platform that offers detailed information on all genetic variants included in the TPMI SNP arrays, such as MAF among TPMI participants, and variant data from reputable sources like ClinVar, OMIM, and the NCBI dbSNP database. (3) TPMI DataView (https://dataview.ibms.sinica.edu.tw/). This platform provides statistical insights on TPMI participants, including data on specific health conditions, lab test results, prescribed medications, and treatments received. (4) TPMI Data Analysis Platform (TDAP). Authorized researchers can access TDAP, a secure central database and analysis platform, once the TPMI Data Access Committee approves their research concepts and their protocols have received approval from Institutional Review Boards (IRBs). Source data are provided with this paper.

## Code availability

The code used for data analysis and quality control is available at https://github.com/HsinChouYang/TPMI_Cohort/.
