# Supplementary material for: The Taiwan Precision Medicine Initiative provides a cohort for large-scale studies
Source: Nature. 2025 Oct 15;648(8092):117–27. doi: 10.1038/s41586-025-09680-x (PMC12675286; doi:10.1038/s41586-025-09680-x)
Supplement: Supplementary file 1 — Supplementary Notes 1–6, Supplementary Figs. 1–14, Supplementary Tables 1–6, descriptions for Supplementary Data 1–27 (data supplied separately) and Supplementary References. [file 41586_2025_9680_MOESM1_ESM.doc]

**The Taiwan Precision Medicine Initiative provides a cohort for large-scale studies**

**Supplemental Information**

| **Note S1** | Consent process .………………………..………….……..………………………………………………….. | 3 |
| --- | --- | --- |
| **Note S2** | TPMv1 and TPMv2 custom-designed SNP arrays ………………………………………………. | 3 |
| **Note S3** | TPMI Data Analysis Platform (TDAP) …………………………………………………………………. | 3 |
| **Note S4** | Access to data …………………………………………………………………………………………………… | 4 |
| **Note S5** | Homozygosity analysis ………………………………………………………………………………………. | 4 |
| **Note S6** | GWASs for T2D, HbA1c, EHT, SBP, and DBP………………………………………………………. | 5 |
| **Figure S1** | TPMI overview and organization structure .………………………..………….…….………….. | 6 |
| **Figure S2** | Timeline and milestones of TPMI .………………………..………….……..………………………… | 7 |
| **Figure S3** | Shared SNPs in TPMv1 and TPMv2 arrays …………………………………………….…………… | 8 |
| **Figure S4** | Data types in the TPMI Data Access Platform (TDAP) ……………………….………………. | 9 |
| **Figure S5** | Population structure of Taiwan’s indigenous groups ………………………………………… | 10 |
| **Figure S6** | Genetic admixture analysis using ADMIXTURE ..………………………………………………… | 11 |
| **Figure S7** | Homozygosity analysis ……………………………………………………………………………………… | 12 |
| **Figure S8** | Sample size evaluation and examples for GWAS and QTL mappings ………………… | 13 |
| **Figure S9** | Data quality control of samples and SNPs for GWAS ………………………………............ | 14 |
| **Figure S10** | Quality control check for missing rate, homozygosity rate, and divergent ancestry ………………………………………………………………………………..……………….………… | 15 |
| **Figure S11** | Manhattan plots for a GWAS of Essential Hypertension and two blood pressures with a covariate adjustment for age, sex, and BMI ….……………………….. | 16 |
| **Figure S12** | Sensitivity analyses considering different SBP cutoffs for a GWAS of Essential Hypertension (EHT) ….………………………………………………………………………………………. | 17 |
| **Figure S13** | Manhattan plots for REGENIE analysis of T2D, HbA1c, EHT, and two blood pressures (SBP and DBP) …….…………………………………………………………………………….. | 18 |
| **Figure S14** | Polygenic risk score analysis based on T2D PGS (PGS002308) …………………………… | 19 |
| **Table S1** | SNP content of TPMv1 and TPMv2 arrays …………………………………………………………. | 20 |
| **Table S2** | Minor allele frequency (MAF) distribution ……………………………………………………….. | 21 |
| **Table S3** | Comparison of minor allele frequency (MAF) between TPMI and other Han Chinese projects ………………………………………………………………………………………………. | 22 |
| **Table S4** | Electronic medical record data in the TPMI Data Access Platform (TDAP) ………… | 23 |
| **Table S5** | Pairwise Fst values among the 10 inferred subgroups (admixed populations) ….. | 24 |
| **Table S6** | 83 genetic conditions for Return of Results (ROR) ……………………………………………. | 25 |
| **Data S1** | Timeline and milestone of TPMI ……………………………………………………………………….. | 27 |
| **Data S2** | Shared SNPs in TPMv1 and TPMv2 arrays ………………………………………………………… | 27 |
| **Data S3** | Population structure of Taiwan’s indigenous groups ……………………………………….. | 27 |
| **Data S4** | Genetic admixture analysis using ADMIXTURE–Cross-validation error across different K values ……………………………………………………………………………………………… | 27 |
| **Data S5** | Genetic admixture analysis using ADMIXTURE–Incremental improvements in log-likelihood across different K values …………………………………………………………….. | 27 |
| **Data S6** | Genetic admixture analysis using ADMIXTURE–Hierarchical clustering dendrogram of the Fst matrix …………………………………………………………………………… | 27 |
| **Data S7** | Homozygosity analysis–Heatmap of homozygosity rate …………………………………… | 27 |
| **Data S8** | Homozygosity analysis–Violin plots of homozygosity rate ………………………………… | 27 |
| **Data S9** | Sample size evaluation and examples for GWAS and QTL mappings–Sample size calculation for a case-control study ………………………………………………………………….. | 27 |
| **Data S10** | Sample size evaluation and examples for GWAS and QTL mappings–Sample size calculation for quantitative trait locus (QTL) study …………………………………………… | 28 |
| **Data S11** | Sample size evaluation and examples for GWAS and QTL mappings–Miami plot of the GWAS for Type 2 Diabetes (T2D) and QTL mapping for HbA1c ………………. | 28 |
| **Data S12** | Manhattan plots for a GWAS of Essential Hypertension and two blood pressures–Essential Hypertension (EHT, 71,548 cases and 130,561 controls) ….. | 28 |
| **Data S13** | Manhattan plots for a GWAS of Essential Hypertension and two blood pressures–Systolic blood pressure (SBP, n = 241,667) ………………………………………. | 28 |
| **Data S14** | Manhattan plots for a GWAS of Essential Hypertension and two blood pressures–Diastolic blood pressure (DBP, n = 241,646) ……………………………………. | 28 |
| **Data S15** | Sensitivity analyses considering different SBP cutoffs for a GWAS of Essential Hypertension–Essential Hypertension: Definition 2 ………………………………………….. | 28 |
| **Data S16** | Sensitivity analyses considering different SBP cutoffs for a GWAS of Essential Hypertension–Essential Hypertension: Definition 3 ………………………………………….. | 28 |
| **Data S17** | Detailed GWAS results ……………………………………………………………………………………… | 28 |
| **Data S18** | Citation for the significant SNPs identified by the TPMI and replicated by other biobank studies ………………………………………………………………………………………………… | 29 |
| **Data S19** | Functional annotation of novel association signals identified in our GWAS for T2D …………………………………………………………………………………………………………………… | 29 |
| **Data S20** | Gene set enrichment and pathway analysis for novel SNP-associated genes identified in our GWAS for T2D using Ingenuity Pathway Analysis (IPA) …………… | 29 |
| **Data S21** | Manhattan plots for REGENIE analysis of T2D, HbA1c, EHT, and two blood pressures (SBP and DBP)–GWAS of Type 2 Diabetes (T2D) (n = 52,290 cases and 192,817 controls) ………………………………………………………………………………………. | 29 |
| **Data S22** | Manhattan plots for REGENIE analysis of T2D, HbA1c, EHT, and two blood pressures (SBP and DBP)–QTL mapping of Hemoglobin A1c (HbA1c) (n = 140,259) …………………………………………………………………………………………………………. | 29 |
| **Data S23** | Manhattan plots for REGENIE analysis of T2D, HbA1c, EHT, and two blood pressures (SBP and DBP)–GWAS of Essential Hypertension (EHT) (n = 71,548 cases and 130,561 controls) ……………………………………………………………….. | 29 |
| **Data S24** | Manhattan plots for REGENIE analysis of T2D, HbA1c, EHT, and two blood pressures (SBP and DBP)–QTL mapping of systolic blood pressure (SBP) (n = 241,667) ……………………………………………………………………………………………………………. | 30 |
| **Data S25** | Manhattan plots for RFGENIE analysis of T2D, HbA1c, EHT, and two blood pressures (SBP and DBP)–QTL mapping of diastolic blood pressure (DBP) (n = 241,646) …………………………………………………………………………………………………………. | 30 |
| **Data S26** | Polygenic risk score analysis based on T2D PGS (PGS002308)–Area under the receiver operating characteristic curve (AUC) …………………………………………………… | 30 |
| **Data S27** | Polygenic risk score analysis based on T2D PGS (PGS002308)–Dose-response effect of PRS levels on the odds ratio of T2D …………………………………………………….. | 30 |
| **References** | ………………………………………………………………………………………………………………………….. | 31 |

**Supplemental Notes**

**Note S1: Consent process**

Before inviting patients or health check-up participants to join TPMI, physicians, nurses, or research staff at affiliated hospitals explained the informed consent form to ensure participants’ understanding. It was emphasized that participation was entirely voluntary and would not affect their medical rights, regardless of their decision. Participants were also informed that they could withdraw from the study at any time. Participants proceeded with samples and data collection only after signing the informed consent form.

**Note S2: TPMv1 and TPMv2 custom-designed SNP arrays**

In collaboration with the TWB and Thermo Fisher Scientific, TPMI developed the TPMv1 array (686,463 SNPs) specifically for the project. Building on the results from the first year of the project, an updated and optimized TPMv2 array (743,227 SNPs) was subsequently built for the rest of the project. The two TPM arrays were meticulously designed to maximize coverage of the Han Chinese population for genetic studies and to include all previously published disease risk variants. This was done by incorporating (1) the genome-wide imputation grid (GWAS grid) based on the next-generation sequencing (NGS) data from the previous studies^1^, which contained approximately 3,000 samples, including common variants and genetic variants with low minor allele frequencies (MAF) ranging from 1% to 5%; and (2) known disease risk variants from databases such as the GWAS Catalog^2^, ACMG^3^, ClinVar^4^, PharmGKB^5^, and OMIM^6^. After evaluating the performance of the TPMv1 array with data from approximately 100,000 individuals, the TPMv2 array was developed by removing markers with very low MAFs in the Taiwanese population to improve genotyping accuracy on the platform. Additionally, markers used to detect known copy number variations (CNVs), loss of function (LOF), and insertion and deletion (INDEL) were added to the array to enhance its utility for genetic analysis (**Table S1**). The TPMv1 and TPMv2 arrays share approximately 495,000 SNPs (**Fig. S3** and **Data S2**). Minor allele frequency distribution is provided (**Table S2**). Genotype concordance was assessed in 1,435 TWB samples genotyped with both TPMv1 (also known as TWBv2.0) and TPMv2 by the National Center for Genome Medicine, using whole-genome sequencing (WGS) data ^1^ as the reference and 401,710 high-quality variants identified ^7^. The average concordance rate between the two arrays was 0.995, with a standard deviation of 0.005.

**Note S3: TPMI Data Analysis Platform (TDAP)**

The TPMI genetic and EMR data are encrypted and stored in a centralized, secure research database at the Academia Sinica that is isolated from the internet. The Academia Sinica Information Technology Team developed the TDAP in a protected computing workplace where all data analyses are conducted exclusively at the workplace by the TPMI Consortium members under supervision by the Academia Sinica IT staff and surveillance camera monitoring to ensure that the participants’ data never leave the database. Only summary results from the analyses are disseminated as approved by the TPMI Publication Committee. TDAP enables users to efficiently retrieve data from the TPMI Data Lake for analysis. Authorized researchers can access TDAP, a secure central database and analysis platform, once their research concepts are approved by the TPMI Data Access Committee and their protocols receive approval from Institutional Review Boards (IRBs). To further support researchers in data exploration, TPMI has developed several platforms. *PheWeb* is adapted from the open-source code released by the University of Michigan PheWeb ^8^ and offers a user-friendly interface for exploring associations between genetic variants and phenotypes, providing access to summary statistics from genome-wide association studies (GWAS) across a wide range of phenotypes and traits. *SNPView* allows users to access information for all loci on the SNP arrays, including the MAF across all TPMI samples, the consistency of loci assessed based on the TWB whole genome sequencing data ^1^, information on genetic variants from ClinVar database ^4^, etc. *DataView* provides information on the number of TPMI participants with specific conditions, laboratory test results, medications prescribed, treatments received, and more.

**Note S4: Access to data**

Access to the TPMI data is based on the consent given by the TPMI participants. First, TPMI researchers registered in TDAP can search metadata and perform initial queries to understand the sample size for specific scientific questions using a system called *PhenoData*, which allows users to input inclusion/exclusion criteria and quickly identify subjects that meet the specified criteria. Researchers can then conduct GWAS and develop polygenic risk scores (PRSs). Upon completion of the analysis, researchers can request to take the summarized results (without any individual information) with them. To date, the TPMI Steering Committee has approved 25 working groups and more than 200 research ideas proposed by TPMI researchers. Researchers outside of the TPMI Consortium with research ideas are encouraged to collaborate with TPMI researchers and pursue studies with the TPMI data.

**Note S5: Homozygosity analysis**

Homozygosity analysis (refer to **Methods–Homozygosity analysis**) reveals that individuals closer to the indigenous and non-East Asian ethnic groups exhibit higher homozygosity (**Fig. S7A** and **Data S7**). The main explanation is that the SNPs on the custom-designed Han Chinese TPM arrays are more often monomorphic in indigenous or non-Han Chinese ancestry groups, also supported by the analysis result based on the SNPs on the TPM arrays, showing that cohorts with an East-Asian ancestry (TPMI, TWB, and EAS) exhibited a lower homozygosity level compared to other non-East-Asian ancestry groups (**Fig. S7B** and **Data S8**). Note that the homozygosity distributions differ from those derived from whole-genome sequencing analysis in other ethnic groups ^9^, serving as a reminder that direct use of the SNP panels on the TPM arrays is well-suited to the genetic research of the East-Asian population but may not be as useful for non-East-Asian ancestry populations. On average, in the TPMI cohort, the median homozygosity rate was 0.801 (standard deviation = 0.003), with 1,197 individuals exceeding six times the interquartile range.

**Note S6: GWASs for T2D, HbA1c, EHT, SBP, and DBP**

The prevalence of diabetes mellitus in Taiwan from 2017-2020 was 11.3%.^10^ The TPMI cohort has 72,659 T2D cases, sufficient to detect T2D-associated SNPs with an odds ratio higher than 1.1 and MAF higher than 0.05. After rigorous data quality control (refer to **Methods–DNA contamination evaluation**, **Methods–Quality control**, and **Figs. S9 – S10**), a preliminary GWAS of T2D (refer to **Methods–GWAS, QTL, and functional annotation**) in the TPMI cohort replicate previous findings such as *Potassium voltage-gated channel subfamily Q member 1* (*KCNQ1*) (*p* = 7.47×10^-122^), *Diabetes-Linked Transcription Factor paired box 4* (*PAX4*) (*p* = 8.57×10^-89^), and *CDKN2B antisense RNA 1* (*CDKN2B-AS1*) (*p* = 3.58×10^-87^) for T2D (n = 52,290 cases and 192,817 controls, top panel in **Fig. S8C** and **Data S11**).

In addition, regarding quantitative trait locus (QTL) analysis, the TPMI cohort has 192,701 participants with HbA1c records, sufficient to detect HbA1c-associated QTLs with a beta coefficient higher than 1.1 and MAF higher than 0.05. Preliminary GWAS of HbA1c identified the same genes in the GWAS for T2D, such as *KCNQ1* (*p* = 1.74×10^-117^), *CDKN2B-AS1* (*p* = 2.16×10^-93^), and *PAX4* (*p* = 1.25×10^-78^), and the additional genes, such as *fructosamine 3 kinase related protein* (*FN3KRP*) (*p* = 1.62×10^-98^) and *chaperonin containing TCP1 subunit 3* (*CCT3*) (*p* = 1.14×10^-21^) (n = 140,259, bottom panel in **Fig. S8C** and **Data S11**).

In another example, GWAS for essential hypertension (EHT defined by the ICD10-code I10, SBP ≥120 mmHg, or DBP ≥80 mmHg; n = 71,548 cases and 130,561 controls, **Fig. S11A** and **Data S12**) and QTL mappings for systolic blood pressure (SBP; n = 241,667, **Fig. S11B** and **Data S13**) and diastolic blood pressure (DBP; n = 241,646, **Fig. S11C** and **Data S14**) simultaneously identified *Fibroblast Growth Factor 5* (*FGF5*) (*p* = 2.25×10^-49^), *5’-nucleotidase, cytosolic II* (*NT5C2*) (*p* = 1.23×10^-21^), and *RAL Guanine Nucleotide Dissociation Stimulator Like 3* (*RGL3*) (*p* = 2.46×10^-10^), along with other genes specific to each of the three GWAS and QTL mappings. Using a lower blood pressure cutoff helps identify variants linked to early or pre-hypertension, supporting earlier intervention.

Sensitive analyses that consider a higher SBP threshold for EHT were conducted. A GWAS for EHT (SBP ≥130 mmHg; n = 70,884 cases and 149,639 controls, **Fig. S12A** and **Data S15**) and a GWAS for EHT (SBP ≥140 mmHg; n = 68,788 cases and 162,337 controls, **Fig. S12B** and **Data S16**) obtained reasonably consistent results across the three SBP cutoffs, illustrating the robustness of the GWASs. Compared to the GWAS for EHT based on a cutoff of 120 mmHG (Top panel in **Fig. S11A** and **Data S12**), the GWAS based on an SBP threshold of 130 mmHg additionally identified *C6orf10* (*p* = 2.41×10^-09^), *LRRC10B* (*p* = 1.85×10^-11^), *TBX3* (*p* = 7.00×10^-10^), and *KLF13* (*p* = 4.64×10^-09^) (**Fig. S12A** and **Data S15**). Similarly, the GWAS based on an SBP threshold of 140 mmHg additionally identified *CACNA1D* (*p* = 8.51×10^-09^), *C6orf10* (*p* = 1.52×10^-10^), *LRRC10B* (*p* = 1.57×10^-11^), and *TBX3* (*p* = 3.30×10^-10^) (**Fig. S12B** and **Data S16**). By incorporating multiple SBP cutoffs, we provide a more comprehensive genetic landscape of EHT, capturing both mild and severe conditions.

**Supplemental Figures**


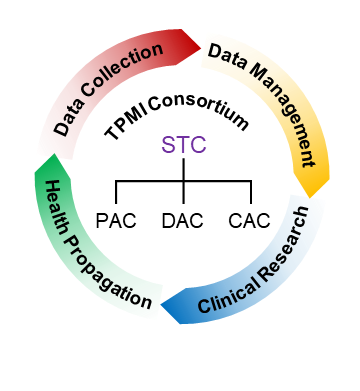


**Figure S1. TPMI overview and organization structure.** The TPMI Consortium is organized by the Steering Committee (STC), the Data Access Committee (DAC), the Clinical Application Committee (CAC), and the Publication Committee (PAC). The TPMI is a consortium led by the Steering Committee, consisting of the principal investigators of the 17 partner organizations (Academia Sinica and 16 medical centers) that make all project-related decisions by consensus. The Steering Committee is assisted by the Data Access Committee (which evaluates research concepts proposed by consortium members and recommends them for Steering Committee approval before data access), the Clinical Application Committee (which establishes best practice health management guidelines based on disease risk), and the Publication Committee (which evaluates abstracts and manuscripts before submission for presentation or publication, respectively). Committee members are drawn from consortium partners and have expertise in clinical research, data analysis, ELSI (Ethical, Legal, and Social Implications), and law. Three teams at the Academia Sinica conduct the study activities: (1) the TPMI Promotion Team coordinates participant recruitment at the partner medical centers, (2) the Genotyping Team performs the genotyping experiments together with six hospital-based genotyping teams at consortium hospitals, and (3) the Statistics and Information Technology Team builds the TPMI Data Access Platform (TDAP) and maintains the central database (i.e., TPMI Data Lake) and assist all researchers in data analysis. In the cyclic workflow of TPMI, Data Collection involves enrolling study participants, collecting genetic and EMR data, and developing population-optimized SNP arrays. Data Management includes data quality control and the establishment of the TDAP and Data Lake. Clinical Research encompasses the establishment of working groups, the proposal of research ideas, the development of polygenic risk score (PRS) algorithms, and advanced genetic analyses. Health Propagation involves the return of results (ROR), engaging in public education initiatives, and closely collaborating with non-profit organizations.

|  |
| --- |
| **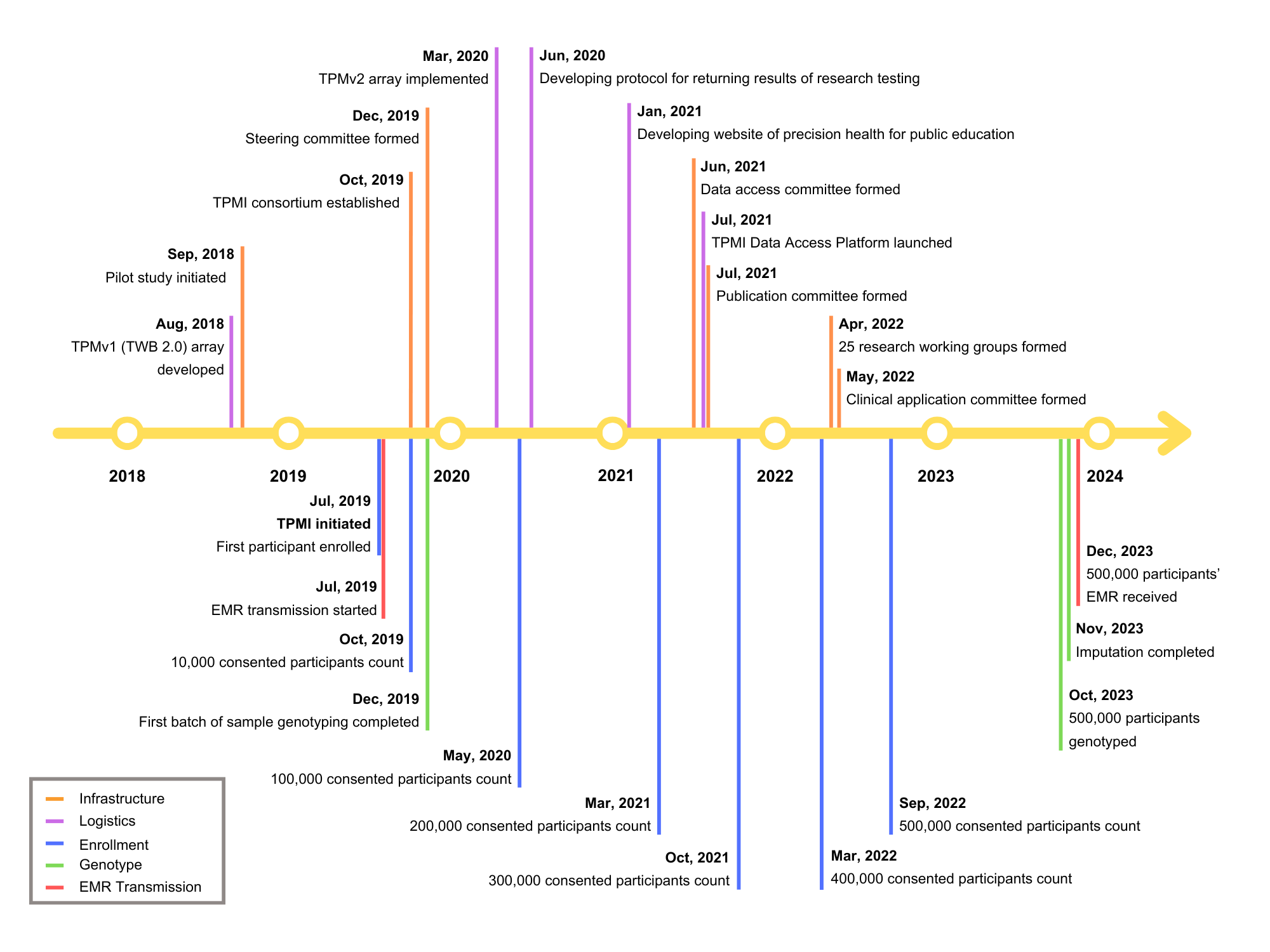** |

**Figure S2. Timeline and milestones of TPMI.** The TPMI was launched officially in July 2019, when the first participant enrolled with institutional review board (IRB) approval. The TPMI Consortium was established in October 2019, with the Steering Committee constituted at the first consortium meeting in December 2019. Soon after the enrollment reached 200,000 in March 2021, the TPMI Data Access Platform (TDAP) was established, and the dataset was made accessible to consortium members in July 2021. The Data Access and Publication Committees were created to facilitate consortium-wide studies and the dissemination of results. The Clinical Application Committee was formed in May 2022 to guide the return of results (ROR) to the participants, formulate risk-based healthcare guidelines, and design research studies to validate the results. A SNP array (TPMv1) co-developed with the Taiwan Biobank (TWB) was used initially, and an updated SNP array (TPMv2) was designed and implemented in March 2020. Approximately 3 years after the first enrollment, 500,000 participants were enrolled by September 2022. As of the end of December 2023, there were 565,390 enrolled participants, of which EMRs from 500,081 participants were transferred from the hospital to the TPMI database after the genotyping of 508,912 participants was completed two months before. Data are provided (**Data S1**).

**Figure S3. Shared SNPs in TPMv1 and TPMv2 arrays.** TPMI developed two custom-designed SNP arrays, TPMv1 and TPMv2. TPMv1 interrogates 686,463 SNPs (represented by green bars), and TPMv2 interrogates 743,227 SNPs (represented by blue bars). The two arrays share 494,968 SNPs (represented by red bars), including 481,987 on autosomes, 10,490 on the X chromosome, 2,171 on the Y chromosome, and 320 on the mitochondrial chromosome. Data are provided (**Data S2**).


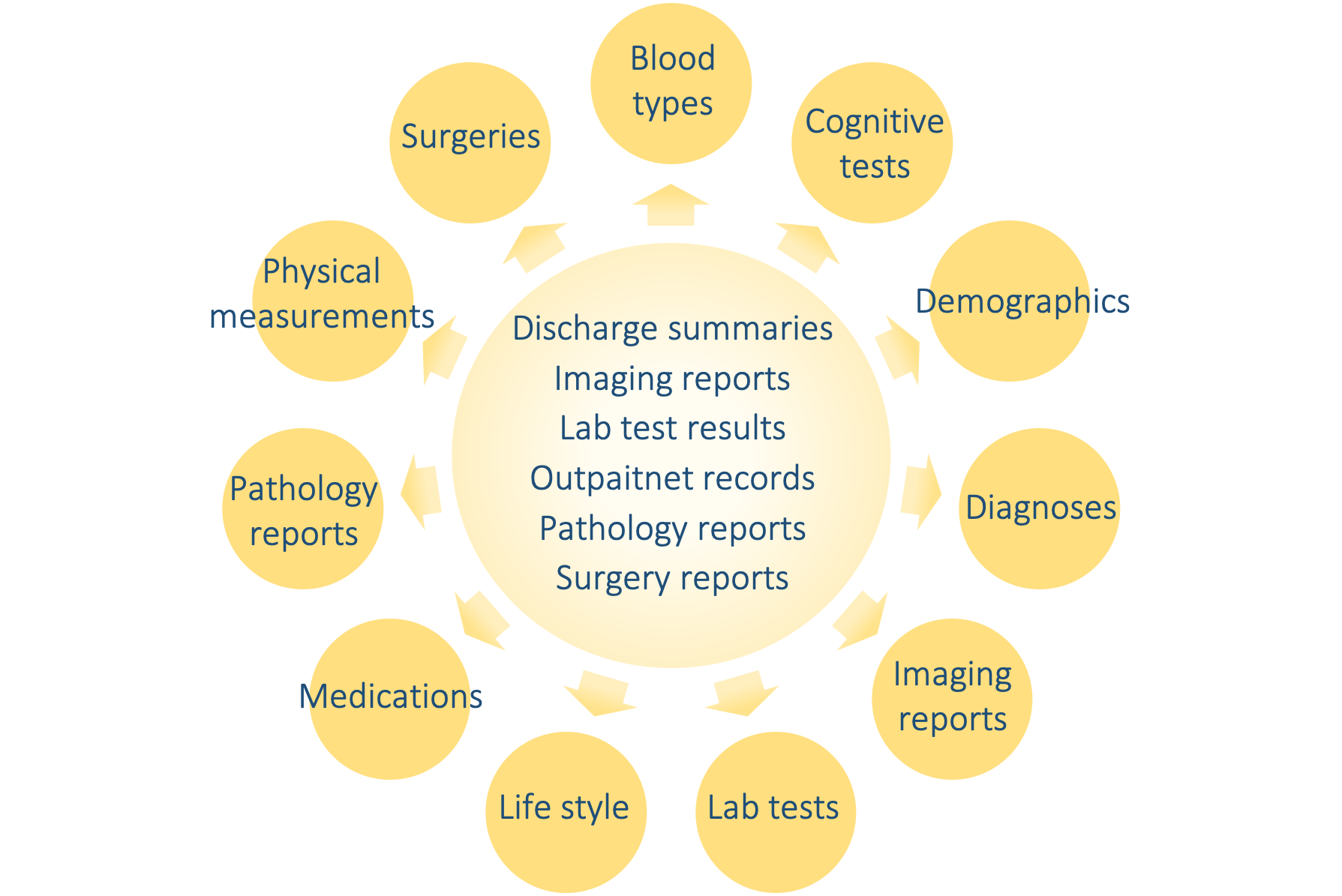


**Figure S4. Data types in the TPMI Data Access Platform (TDAP).** TPMI collected electronic medical records (EMR) data, which includes discharge summaries, imaging reports, lab test results, outpatient records, pathology reports, and surgery reports. Each type of record comprises both free-text sections and predefined structured data. The data can be further divided into the following subcategories: blood types, cognitive tests, demographics, diagnoses, imaging reports, lab tests, lifestyle, medications, pathology reports, physical measurements, and surgeries, totaling more than 140 EMR variables.

**(A)**

**
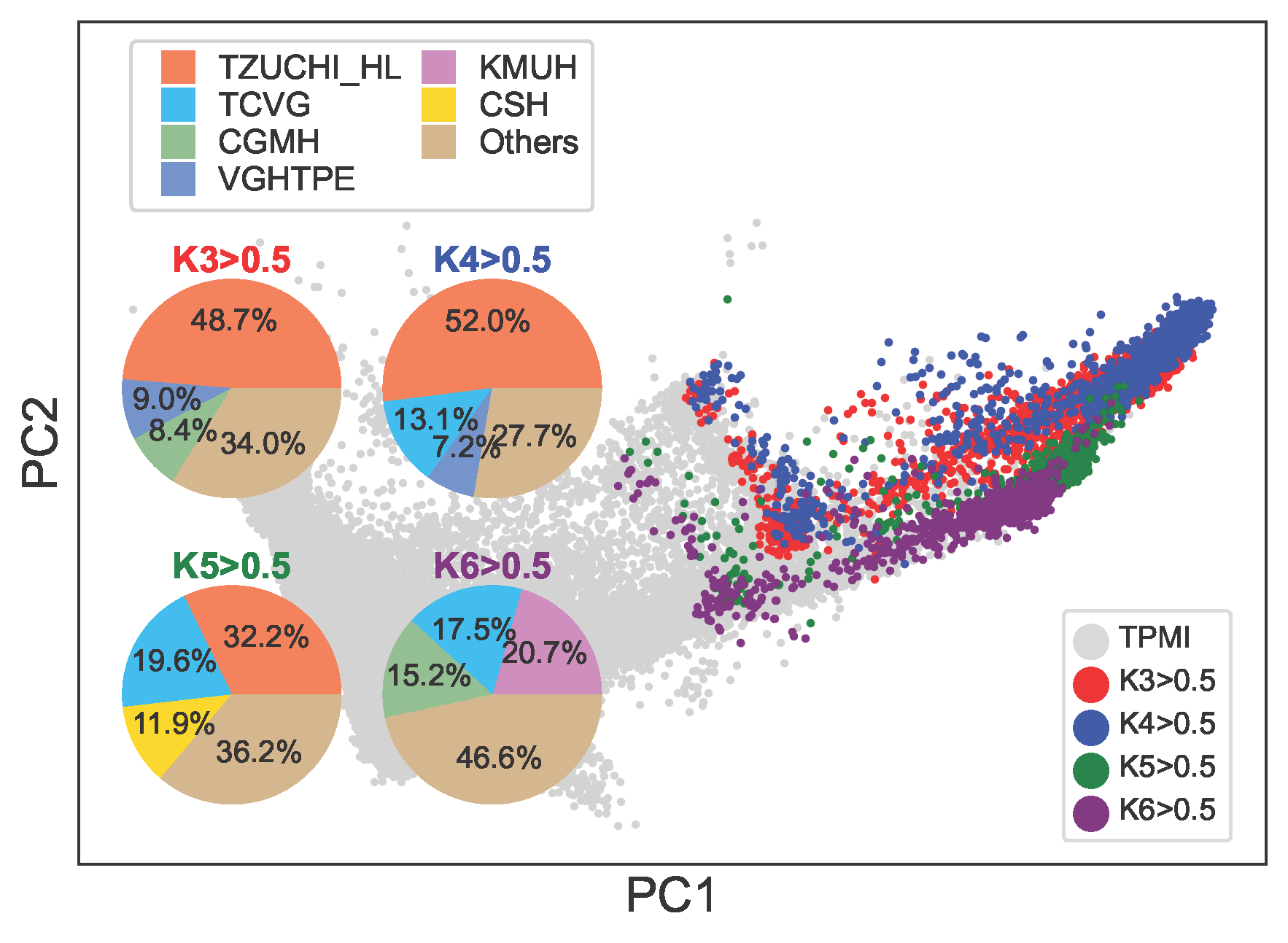
**

**(B)**


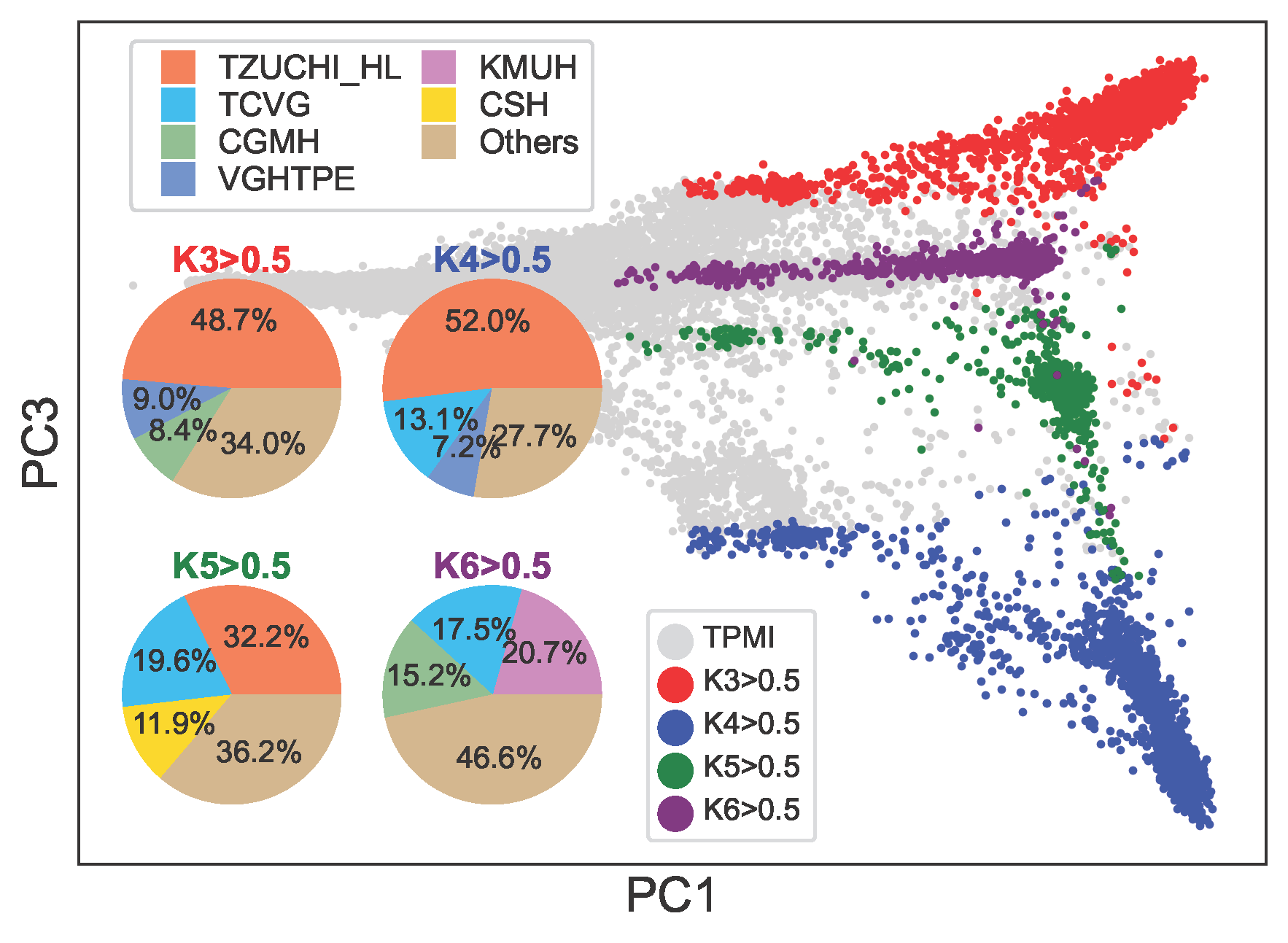


**Figure S5. Population structure of Taiwan’s indigenous groups.** Our population genetic structure analysis reveals that K3–K6 represent Taiwan’s indigenous groups. For each indigenous group, we identify the top three medical centers with the largest sample size: Hualien Tzu Chi Hospital (TZUCHI_HL), Linkou Chang Gung Memorial Hospital (CGMH), and Taichung Veterans General Hospital (TCVG). For example, approximately half of the participants classified as K3 and K4 were recruited by Hualien Tzu Chi Hospital, the largest medical center in eastern Taiwan. This hospital serves as a major healthcare provider for indigenous populations in the region, as indicated by demographic statistics from the Council of Indigenous Peoples (<https://www.cip.gov.tw/en/index.html>) and the Department of Household Registration, Ministry of the Interior, Taiwan (<https://www.ris.gov.tw/app/en>). **(A) PCA plot of PC1 vs. PC2**. **(B) PCA plot of PC1 vs. PC3**. Data are provided (**Data S3**).


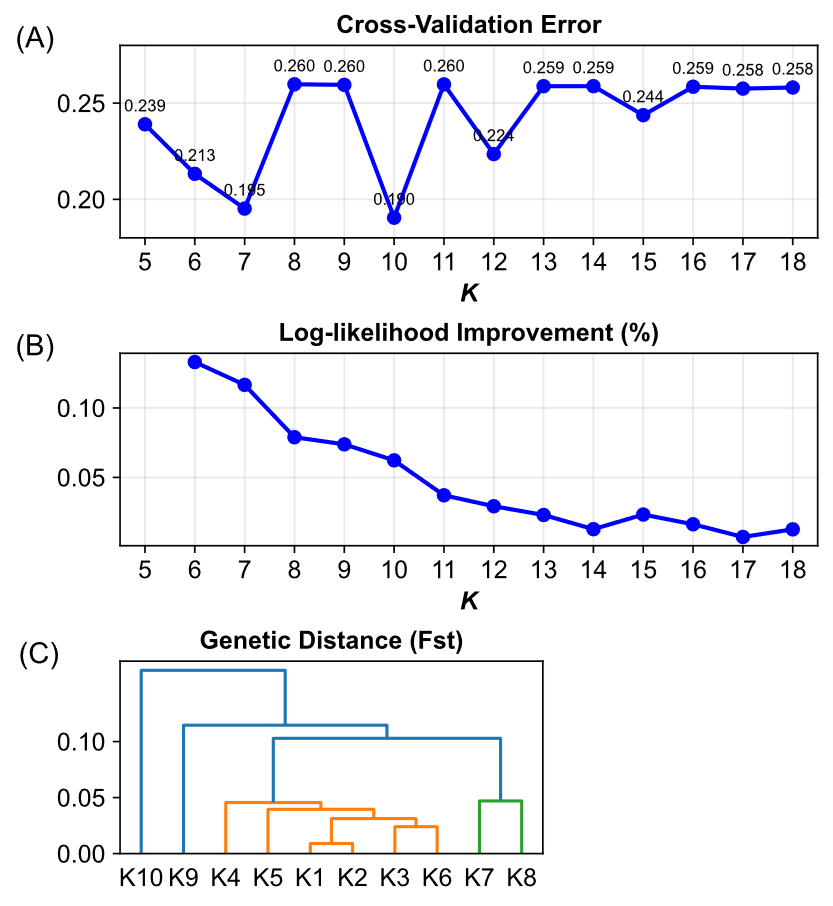


**Figure S6. Genetic admixture analysis using ADMIXTURE. (A) Cross-validation error across different *K* values**. The optimal number of ancestral populations (*K*) was determined using ADMIXTURE’s cross-validation procedure, evaluating values from *K*=5 to *K*=18, with *K*=10 showing the lowest cross-validation error (0.190). Data are provided (**Data S4**). **(B) Incremental improvements in log-likelihood across different *K* values**. We observed that the improvement in log-likelihood stabilized after *K* > 12, which discouraged consideration of large *K* values and supported both *K* = 7 (a local CV error minimum) and *K* = 10 (the global CV error minimum) as reasonable choices. However, *K* = 7 exhibits a higher CV error and merges biologically distinct groups. Considering both statistical evidence and biological plausibility, we determined *K* = 10 as the most appropriate representation of population structure for this dataset. Data are provided (**Data S5**). **(C) Hierarchical clustering dendrogram of the Fst matrix**. Apart from comparisons involving non–East Asian ancestry groups (*K*7–*K*10), the genetic differentiation among Han Chinese ancestry subgroups (*K*1–*K*2) and Taiwan’s indigenous-enriched admixed groups (*K*3–*K*6) is modest, as reflected in their relatively small pairwise Fst values (**Table S5**). A hierarchical clustering dendrogram based on average linkage of the Fst matrix (**Table S5**) was constructed to visualize the genetic relationships among the ten inferred admixed subgroups. This outcome is consistent with known demographic history: admixture between Han Chinese ancestry and Taiwan’s indigenous populations began a few hundred years ago, with the most substantial migration and gene flow occurring after 1945. Given this relatively short evolutionary timeframe, the level of genetic differentiation among subgroups remains limited. In sum, these results support our choice of *K* = 10 as a statistically robust and biologically reasonable model, while acknowledging the shallow genetic structure in this population. Data are provided (**Data S6**).

**Figure S7. Homozygosity analysis. (A) Heatmap of homozygosity rate.** **(B) Violin plots of homozygosity rate.** Data are provided (**Data S7–S8**).

**
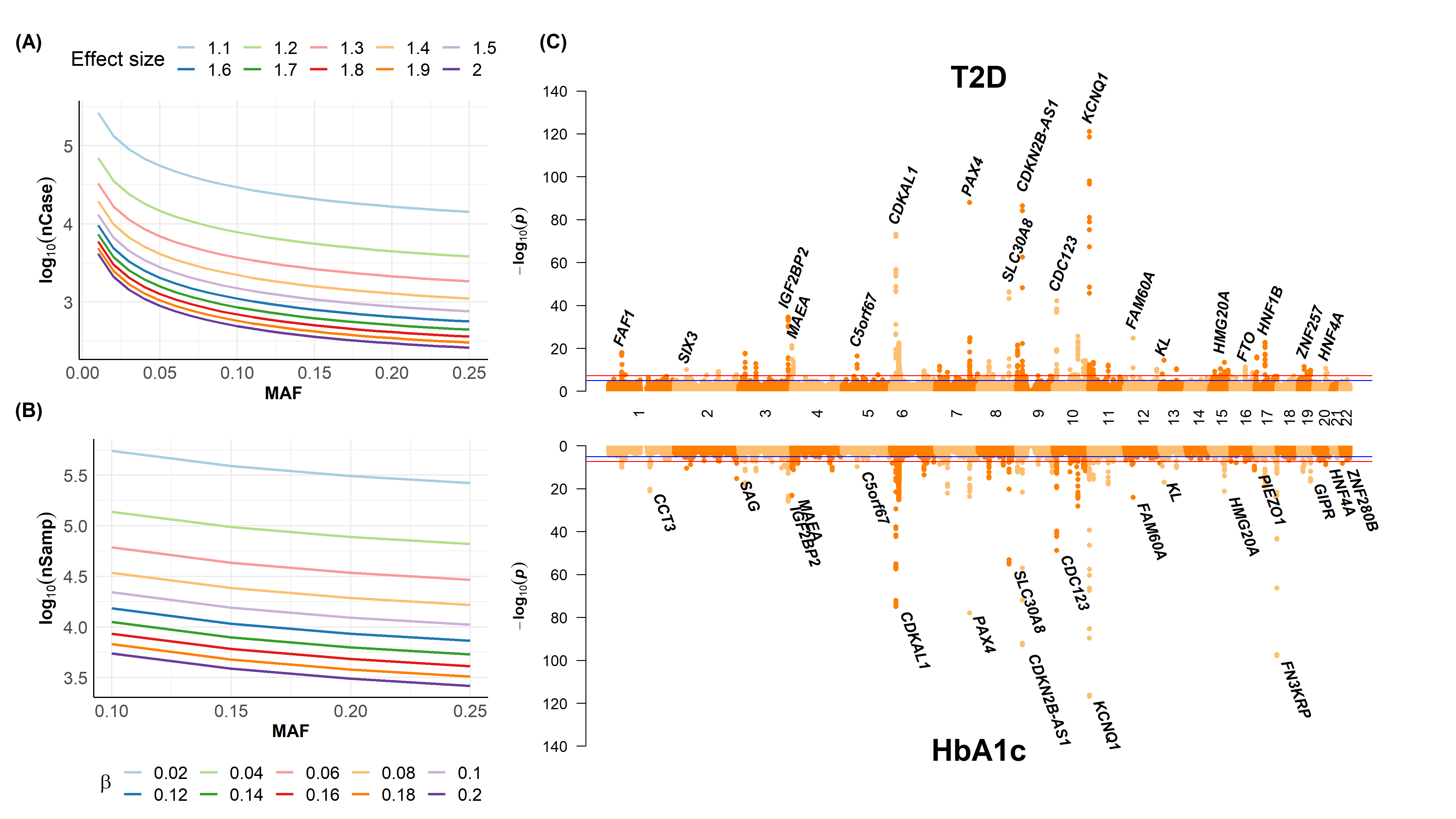
**

**Figure S8. Sample size evaluation and examples for GWAS and QTL mappings.** **(A) Sample size calculation for a case-control study.** The horizontal axis is minor allele frequency (MAF). The vertical axis is the number of cases on a scale of log 10. Curves with different colors reflect different effect sizes. Data are provided (**Data S9**). **(B) Sample size calculation for quantitative trait locus (QTL) study.** The horizontal axis is minor allele frequency (MAF). The vertical axis is the number of participants on a scale of log 10. Curves with different colors reflect different effect sizes (i.e., beta values). Data are provided (**Data S10**). **(C) Miami plot of the GWAS for Type 2 Diabetes (T2D) and QTL mapping for HbA1c.** A Firth logistic regression model was used for the T2D GWAS, and linear regression was used for the HbA1c QTL mapping. All statistical tests were two-sided. Multiple testing adjustment was applied using a genome-wide significance threshold of P < 1e-8. The red (blue) reference line indicates a significance level of *p* = 5×10^-8^ (*p* = 1×10^-5^). SNPs with *p* < 1×10^-8^ were identified, and the names of the genes harboring these significant SNPs are displayed. Data are provided (**Data S11**).


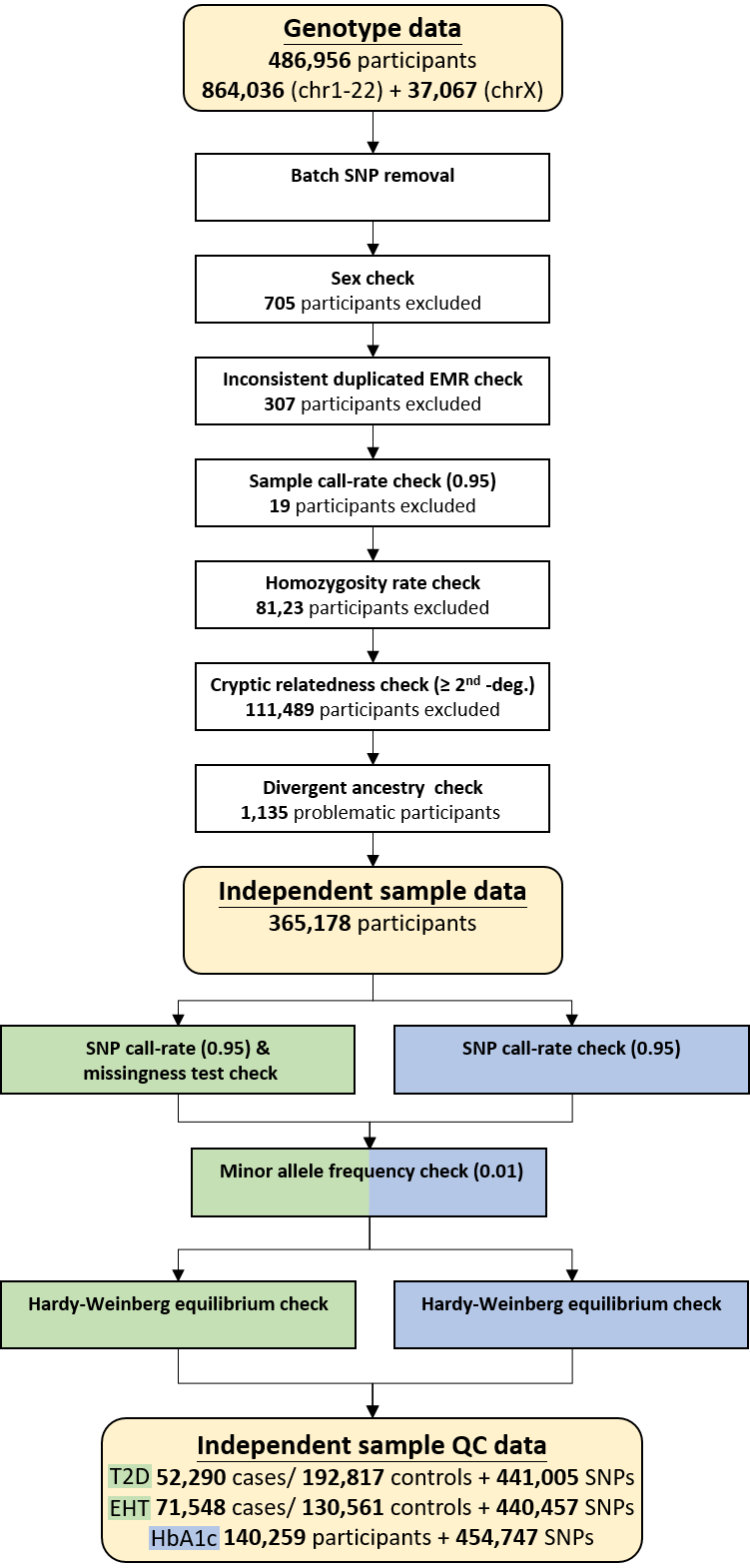


**Figure S9. Data quality control of samples and SNPs for GWAS.**

| **(A)**  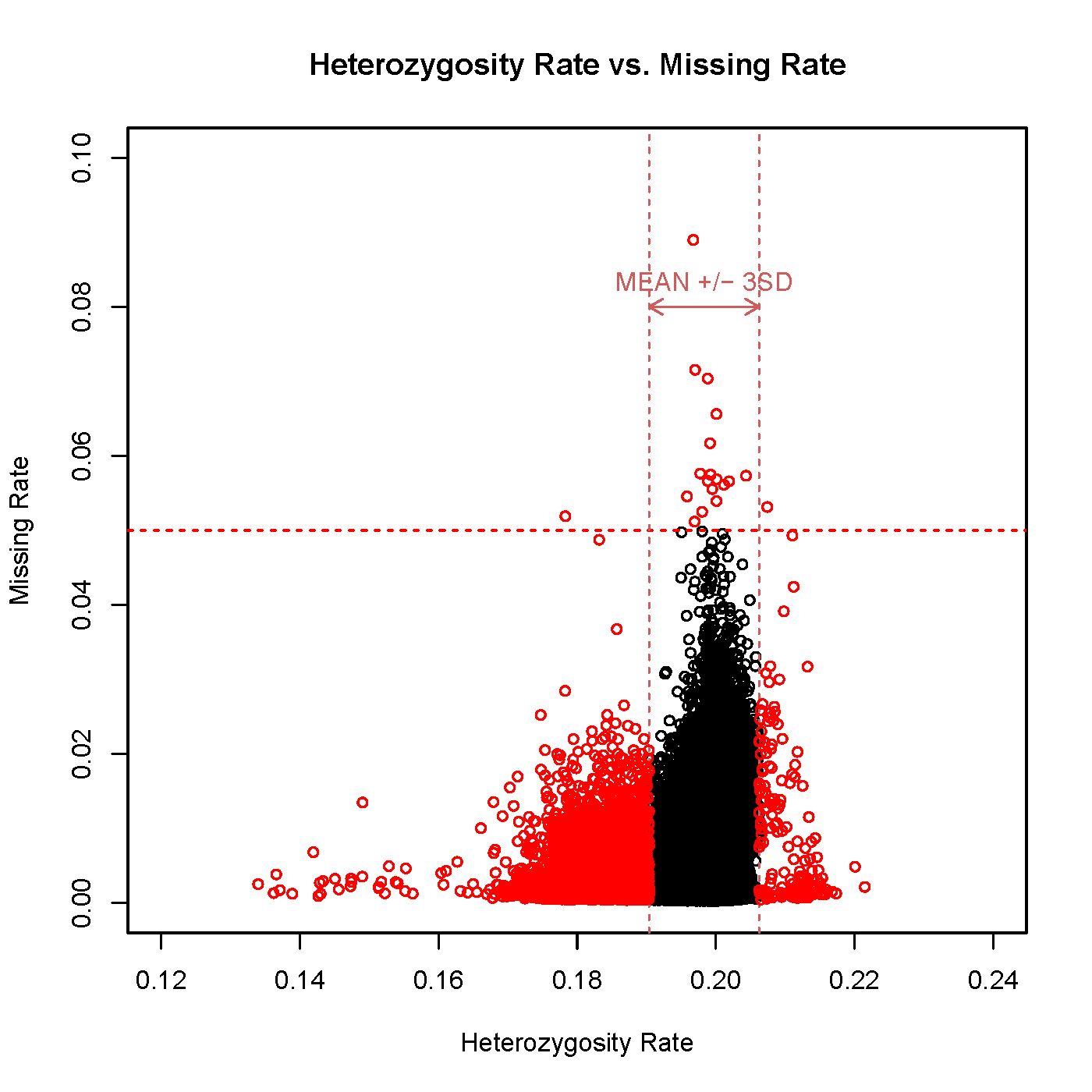 | **(B)**  **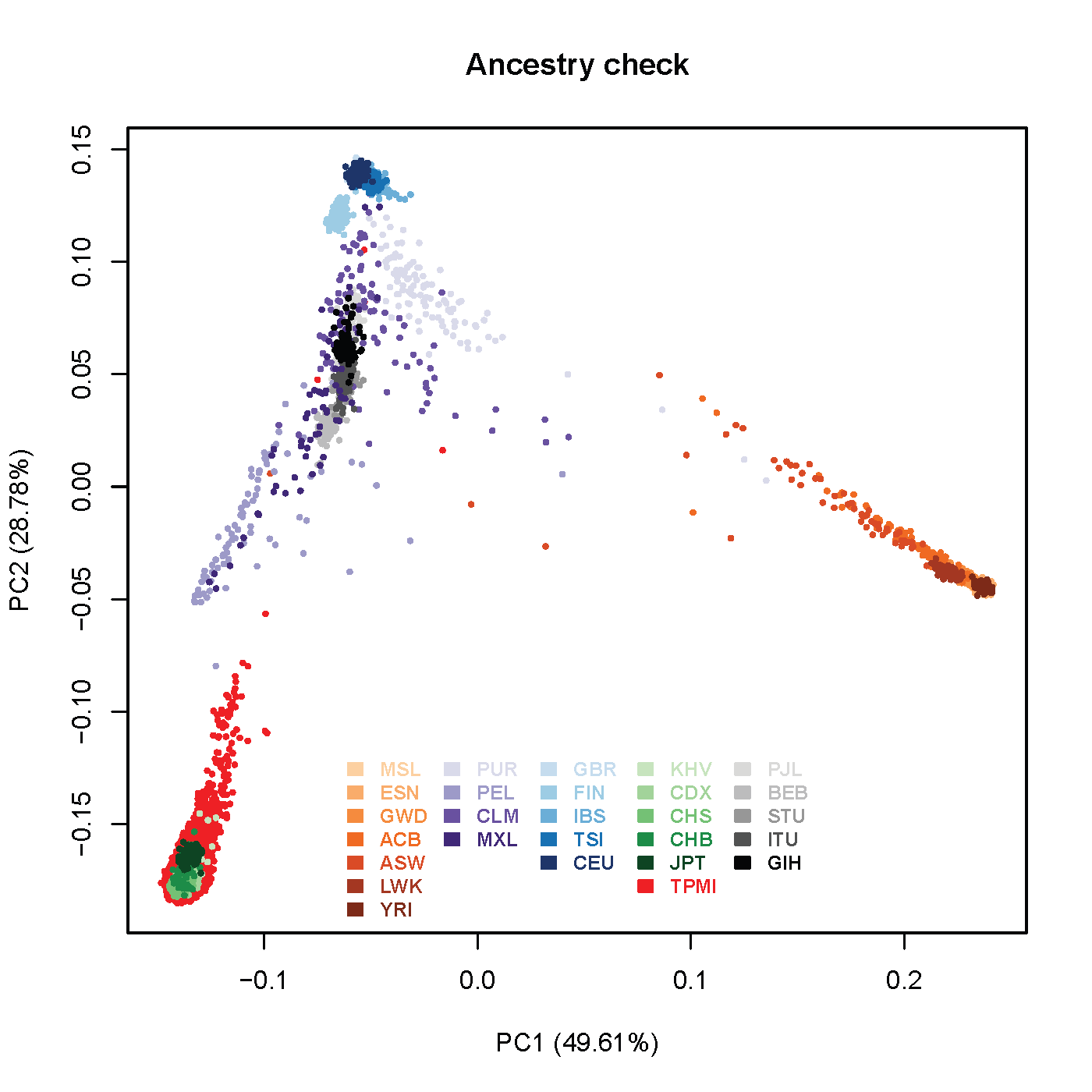** |
| --- | --- |
| **(C)**  **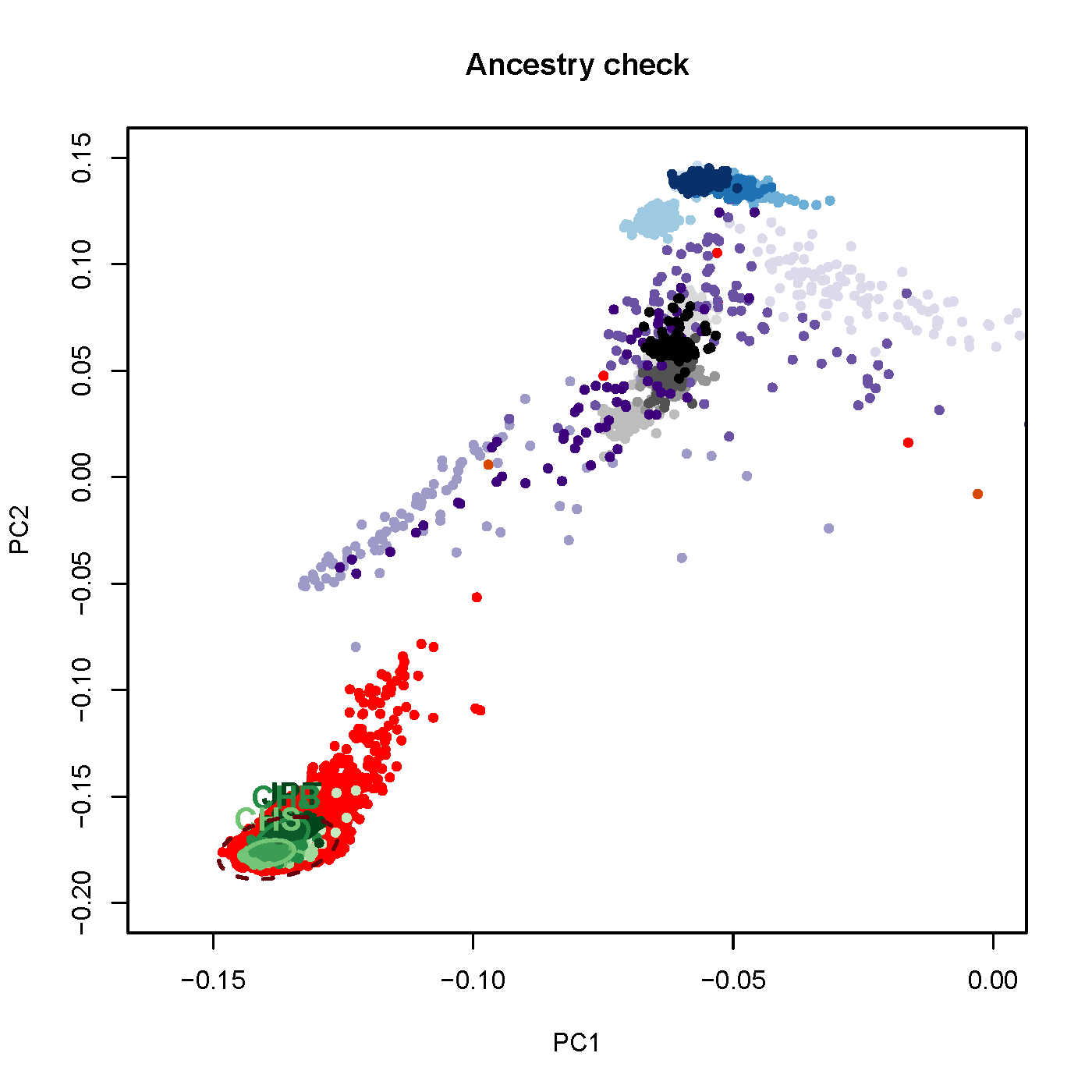** | **(D)**  **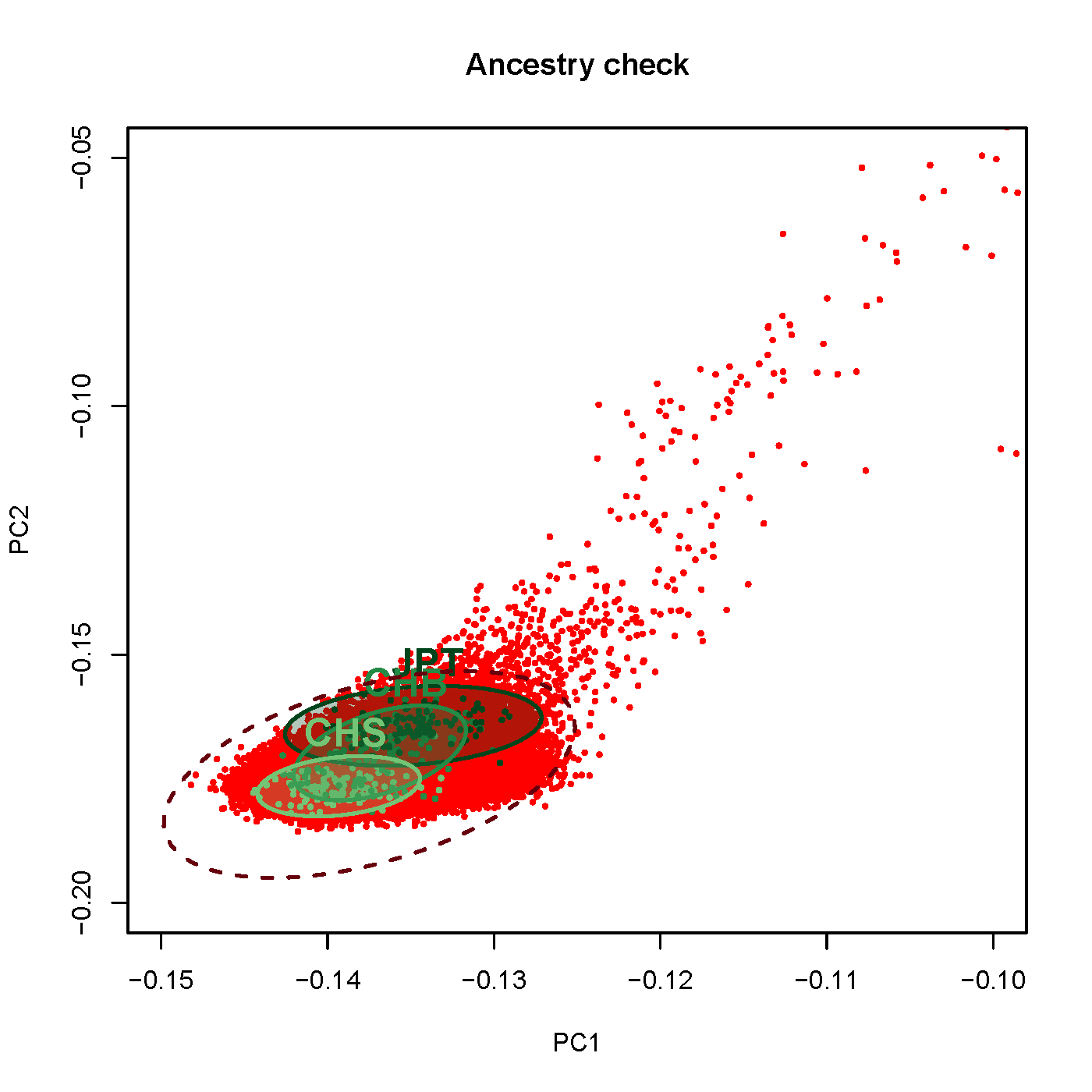** |

**Figure S10. Quality control check for missing rate, homozygosity rate, and divergent ancestry. (A) Quality control check for missing rate and homozygosity rate. (B) Ancestry check using PCA for global populations. (C) Ancestry check using PCA for non-African populations. (D) Ancestry check using PCA for Asian populations.**


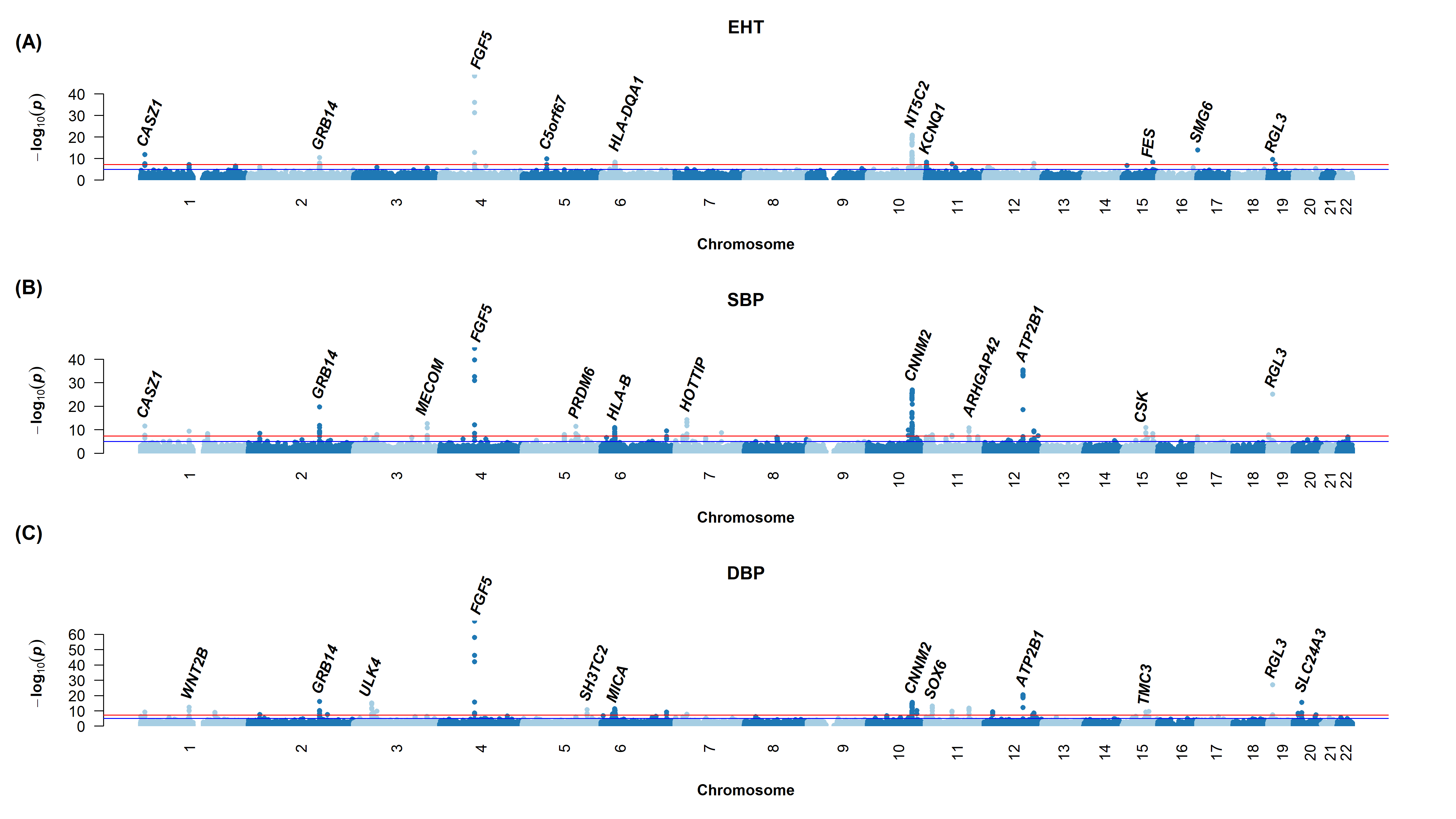


**Figure S11. Manhattan plots for a GWAS of Essential Hypertension and two blood pressures with a covariate adjustment for age, sex, and BMI. (A) Essential Hypertension – Definition 1 (EHT defined by the ICD10-code I10, SBP ≥120 mmHg, or DBP ≥80 mmHg; 71,548 cases and 130,561 controls).** Data are provided (**Data S12**)**; (B) Systolic blood pressure (SBP, n = 241,667).** Data are provided (**Data S13**)**; (C) Diastolic blood pressure (DBP, n = 241,646).** Data are provided (**Data S14**).


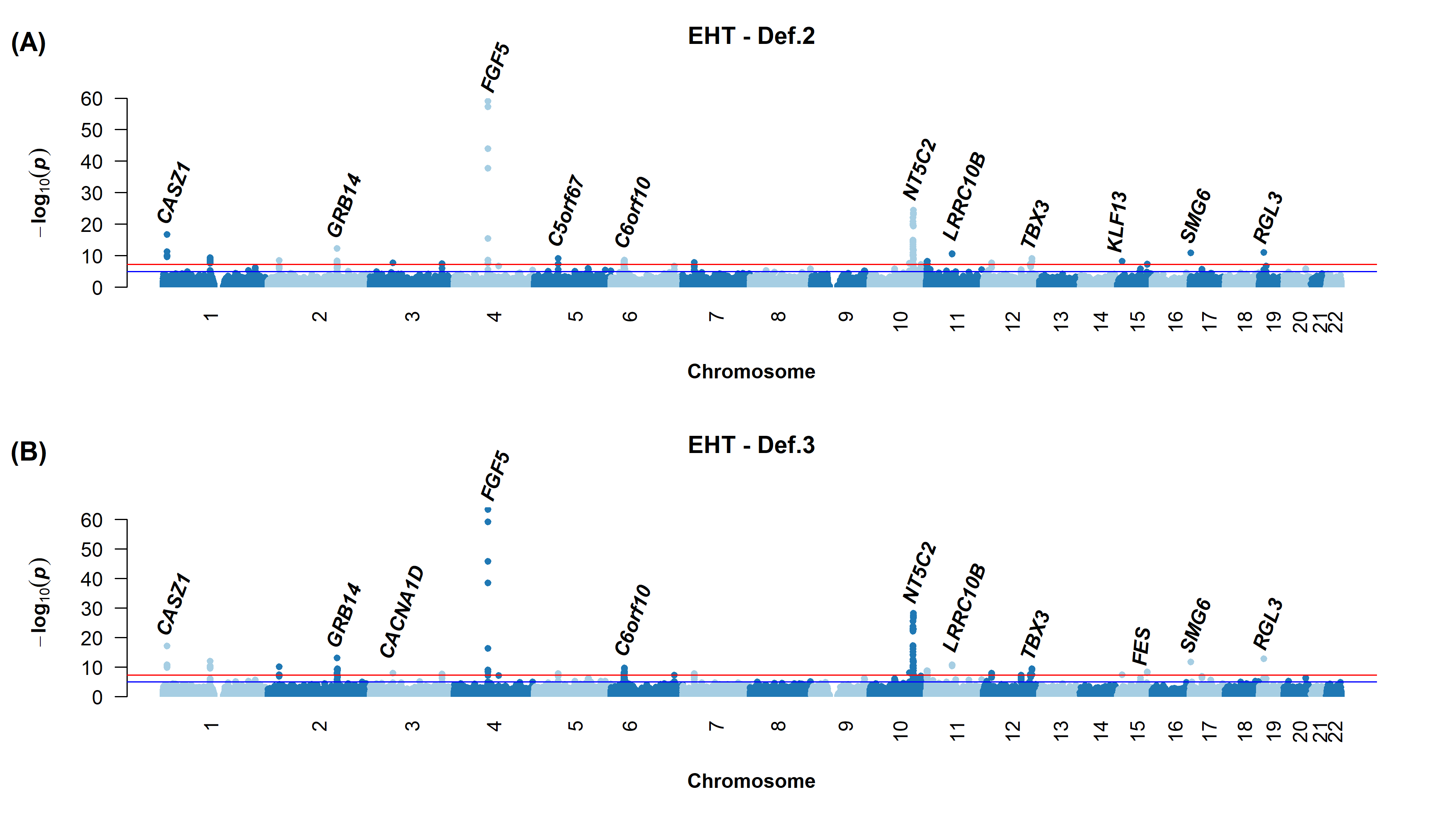


**Figure S12. Sensitivity analyses considering different SBP cutoffs for a GWAS of Essential Hypertension (EHT). (A) Essential Hypertension – Definition 2 (EHT defined by the ICD10-code I10, SBP ≥130 mmHg, or DBP ≥80 mmHg; 70,884 cases and 149,639 controls).** Data are provided (**Data S15**)**; (B) Essential Hypertension – Definition 3 (EHT defined by the ICD10-code I10, SBP ≥140 mmHg, or DBP ≥80 mmHg; 68,788 cases and 162,337 controls).** Data are provided (**Data S16**)


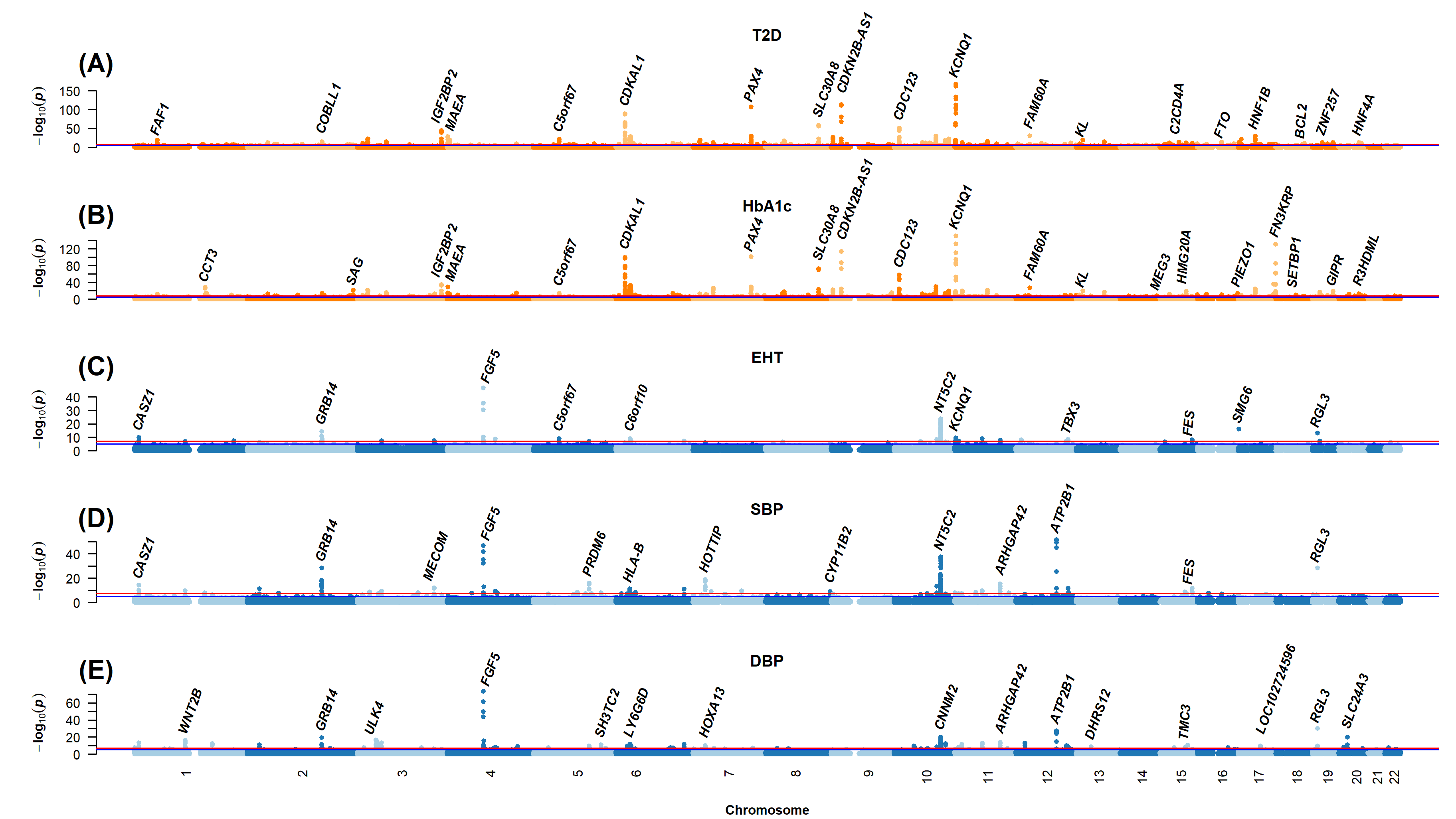


**Figure S13. Manhattan plots for REGENIE analysis of T2D, HbA1c, EHT, and two blood pressures (SBP and DBP). (A) GWAS of Type 2 Diabetes (T2D) (n = 52,290 cases and 192,817 controls).** Data are provided (**Data S21**)**; (B) QTL mapping of Hemoglobin A1c (HbA1c) (n = 140,259).** Data are provided (**Data S22**)**; (C) GWAS of Essential Hypertension (EHT) (n = 71,548 cases and 130,561 controls).** Data are provided (**Data S23**)**; (D) QTL mapping of systolic blood pressure (SBP) (n = 241,667).** Data are provided (**Data S24**)**; (E) QTL mapping of diastolic blood pressure (DBP) (n = 241,646).** Data are provided (**Data S25**).

Specificity

Sensitivity


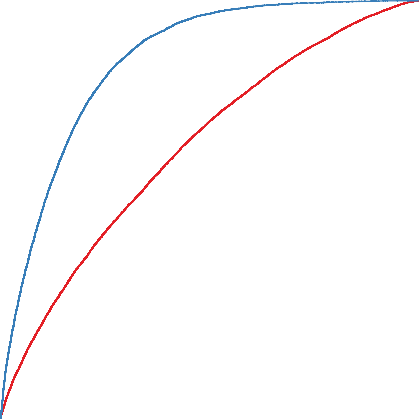


1.0

0.8

0.6

**AUC_PRS_ : 0.6509**

**5**

0.40.20.0

0.0

0.2

0.4

0.6

0.8

1.0

**(A)**

**OR**

**0%−10%**

**10%−20%**

**20%−30%**

**30%−40%**

**40%−60%**

**60%−70%**

**70%−80%**

**80%−90%**

**90%−100%**

**0**

1

2

3

4

**(B)**

**PRS**

**PRS + age + sex + BMI**

**AUC_PRS_: 0.6509**

**AUC_PRS+age+sex+BMI_: 0.8565**

**0.36**

**0.3**

**0.55**

**0.66**

**0.79**

**0.5**

**0.64**

**0.81**

**1.36**

**1.24**

**1.5**

**1.75**

**1.79**

**1.2**

**3.74**

**2.68**

**Figure S14. Polygenic risk score analysis based on T2D PGS (PGS002308).** **(A) Area under the receiver operating characteristic curve (AUC)**. AUC of PRS (red curve) and AUC of PRS, age, sex, and BMI (blue curve) are displayed. Data are provided (**Data S26**). **(B)** **Dose-response effect of PRS levels on the odds ratio of T2D**. Dose-response effect of PRS levels on the odds ratio of T2D (red line) and dose-response effect of a combination of PRS, age, sex, and BMI (blue curve). Data are provided (**Data S27**).

**Supplemental Tables**

**Table S1. SNP content of TPMv1 and TPMv2 arrays.** TPMv1 contains 686,463 SNPs, and TPMv2 contains 743,227 SNPs. Note that some SNPs from different categories may overlap.

| | **Category** | **TPMv1** | **TPMv2** | | --- | --- | --- | | GWAS Grid | 403,082 | 580,364 | | GWAS Catalog | 67,002 | 41,645 | | ACMG | 40,295 | 2,803 | | ClinVar | 90,343 | 15,467 | | PharmGKB | 3,949 | 2,911 | | OMIM | 82,425 | 165,631 | | Loss of function (LoF) | 49,794 | 48,477 | | Copy number variation (CNV) | 42,660 | 22,884 | | Insertion and deletion (INDEL) | 55,724 | 28,311 | |
| --- | --- | --- | --- | --- | --- | --- | --- | --- | --- | --- | --- | --- | --- | --- | --- | --- | --- | --- | --- | --- | --- | --- | --- | --- | --- | --- | --- | --- | --- | --- |

GWAS Catalog: <https://www.ebi.ac.uk/gwas/>

ACMG: https://www.acmg.net/

ClinVar: <https://www.ncbi.nlm.nih.gov/clinvar/>

PharmGKB: https://www.pharmgkb.org/

OMIM: <https://www.omim.org/>

**Table S2. Minor allele frequency (MAF) distribution.**

| MAF | TPMv1 (n = 165,596) | TPMv2 (n = 321,360) |
| --- | --- | --- |
| [0,0.001] | 132,886 | 25,533 |
| (0.001,0.005] | 10,382 | 6,572 |
| (0.005,0.01] | 18,547 | 11,458 |
| (0.01,0.05] | 196,275 | 280,472 |
| (0.05,0.1] | 71,230 | 106,352 |
| (0.1,0.5] | 219,291 | 267,028 |
| Total | 648,611 | 697,415 |

**Table S3. Comparison of minor allele frequency (MAF) between TPMI and other Han Chinese projects. (A) Comparison between TPMI and the China Metabolic Analytics Project (ChinaMAP); (B) Comparison between TPMI and the Westlake BioBank for Chinese (WBBC).** Common variants (C): MAF > 0.05; Low-frequency variants (L): 0.01 < MAF ≤ 0.05; Rare variants (R): MAF ≤ 0.01; Ultra-rare variants (U): MAF < 0.001; <NA> indicates MAF = 0.

| **(A)** TPMI vs. ChinaMAP | |  |  |  | **(B)** TPMI vs. WBBC |  |  |  |
| --- | --- | --- | --- | --- | --- | --- | --- | --- |
| Project | TPMI |  | ChinaMAP |  | Project | TPMI |  | WBBC |
| # of individuals | 483,653 |  | 10,588 |  | # of individuals | 483,653 |  | 4,480 |
| # of loci | 8,046,864 |  | 147,448,941 |  | # of loci | 8,046,864 |  | 80,090,241 |
|  | Variant type in TPMI | Variant type in ChinaMAP |  |  |  | Variant type in TPMI | Variant type in WBBC |  |
|  | <NA> | C | 993,185 |  |  | <NA> | C | 505,403 |
|  | <NA> | L | 518,420 |  |  | <NA> | L | 340,959 |
|  | <NA> | R | 6,090,656 |  |  | <NA> | R | 6,368,992 |
|  | <NA> | U | 132,528,053 |  |  | <NA> | U | 65,983,091 |
|  | **C** | **<NA>** | **499,449** |  |  | **C** | **<NA>** | **944,878** |
|  | C | C | 5,328,217 |  |  | C | C | 4,872,469 |
|  | C | L | 55,052 |  |  | C | L | 48,987 |
|  | C | R | 1,338 |  |  | C | R | 304 |
|  | C | U | 1,303 |  |  | C | U | 18,721 |
|  | **L** | **<NA>** | **191,354** |  |  | **L** | **<NA>** | **175,756** |
|  | L | C | 133,012 |  |  | L | C | 100,647 |
|  | L | L | 1,448,999 |  |  | L | L | 1,485,793 |
|  | L | R | 96,993 |  |  | L | R | 95,091 |
|  | L | U | 2,032 |  |  | L | U | 15,103 |
|  | **R** | **<NA>** | **37,434** |  |  | **R** | **<NA>** | **34,434** |
|  | R | C | 278 |  |  | R | C | 14 |
|  | R | L | 137,571 |  |  | R | L | 137,253 |
|  | R | R | 110,749 |  |  | R | R | 111,841 |
|  | R | U | 3,083 |  |  | R | U | 5,573 |

**Table S4. Electronic medical record data in the TPMI Data Access Platform (TDAP).**

| **Record type** | **Outpatient records** | **Discharge summary** | **Lab test results** | **Pathology reports** | **Surgery reports** | **Imaging reports** |
| --- | --- | --- | --- | --- | --- | --- |
| Free text data | Division, Condition summary, Family history | Division, Chief complaint, Laboratory data, Imaging study, Hospital course, Complications | Reference range, Remarks | Reference range, Remarks | Adverse drug, Blood Transfusion reaction, history of transplantation | Division, Findings, Chief complaint, Results |
| Structured data | Age, Sex, Date, Blood type, Major illness, ICD diagnosis, Procedures, Prescription | Age, Sex, Date, ICD diagnosis, Cancer staging | Age, Sex, Date, NHI code, test results | Age, Sex, Date, Major illness, ICD diagnosis, Specimen site, Result | Age, Sex, Date, Blood type, Pre- / Post-operation ICD diagnosis, Surgical approach, Operative Finding | Age, Sex, Date, Image type, Body areas, ICD diagnosis |

ICD diagnosis is based on ICD-9 or ICD-10.

**Table S5. Pairwise Fst values among the 10 inferred subgroups (admixed populations).** K1 represents North-enriched admixed Han (low PC1, medium PC2) and K2 represents South-enriched admixed Han (low PC1, low PC2). K3–K6 represent Taiwan’s indigenous-enriched admixed groups (high PC1, medium PC2). K7–K10 represents global immigrants (high PC2), with specific ancestry assignments: K7 corresponds to European (EUR) ancestry; K8 corresponds to South Asian (SAS) ancestry; K9 corresponds to American (AMR) ancestry; and K10 corresponds to African (AFR) ancestry.

|  | **K1** | **K2** | **K3** | **K4** | **K5** | **K6** | **K7** | **K8** | **K9** | **K10** |
| --- | --- | --- | --- | --- | --- | --- | --- | --- | --- | --- |
| **K1** | 0 | 0.009 | 0.035 | 0.052 | 0.047 | 0.035 | 0.102 | 0.069 | 0.092 | 0.154 |
| **K2** | 0.009 | 0 | 0.027 | 0.044 | 0.039 | 0.028 | 0.105 | 0.072 | 0.099 | 0.156 |
| **K3** | 0.035 | 0.027 | 0 | 0.042 | 0.036 | 0.024 | 0.121 | 0.088 | 0.117 | 0.172 |
| **K4** | 0.052 | 0.044 | 0.042 | 0 | 0.046 | 0.044 | 0.135 | 0.103 | 0.132 | 0.185 |
| **K5** | 0.047 | 0.039 | 0.036 | 0.046 | 0 | 0.036 | 0.132 | 0.099 | 0.129 | 0.182 |
| **K6** | 0.035 | 0.028 | 0.024 | 0.044 | 0.036 | 0 | 0.121 | 0.088 | 0.118 | 0.172 |
| **K7** | 0.102 | 0.105 | 0.121 | 0.135 | 0.132 | 0.121 | 0 | 0.047 | 0.124 | 0.136 |
| **K8** | 0.069 | 0.072 | 0.088 | 0.103 | 0.099 | 0.088 | 0.047 | 0 | 0.106 | 0.124 |
| **K9** | 0.092 | 0.099 | 0.117 | 0.132 | 0.129 | 0.118 | 0.124 | 0.106 | 0 | 0.191 |
| **K10** | 0.154 | 0.156 | 0.172 | 0.185 | 0.182 | 0.172 | 0.136 | 0.124 | 0.191 | 0 |

**Table S6. 83 genetic conditions for Return of Result (ROR).** **(A) Genetic conditions for health management**; **(B) Genetic conditions for disease diagnosis**; **(C) Pharmacogenetic associations for medicines**.

| |  | **Disease / Medicine** | **Gene** | | --- | --- | --- | | **(A)**  Genetic conditions for health management | Alcohol flushing reaction | *ALDH2* | | Hereditary cancer-predisposing_syndrome/Breast-ovarian cancer, familial 4 | *RAD51D* | | Familial hypercholesterolemia | *APOB, LDLR* | | DFNA 2 Nonsyndromic Hearing Loss | *KCNQ4* | | Hereditary pancreatitis | *PRSS1,SPINK1* | | Cerebral autosomal dominant arteriopathy with subcortical infarcts and leukoencephalopathy (CADASIL) | *NOTCH3* | | Hyperuricemia | *ABCG2* | | **(B)**  Genetic conditions for disease diagnosis | Glucose-6-phosphate dehydrogenase deficiency | *G6PD* | | Meckel syndrome | *MKS1* | | beta Thalassemia | *HBB* | | Total iodide organification defect/Neonatal transient hypothyroidism | *TPO* | | congenital hypothyroidism | *TSHR* | | PMM2-congenital disorder of glycosylation | *PMM2* | | primary carnitine deficiency | *SLC22A5* | | Wilson disease | *ATP7B* | | Joubert Syndrome | *CEP290* | | primary autosomal recessive microcephaly 1 | *MCPH1* | | glutaric acidemia type I | *GCDH* | | Krabbe Disease (Later-onset/adult-onset form) | *GALC* | | citrin deficiency (adult-onset type II citrullinemia) | *SLC25A13* | | sialidosis | *NEU1* | | Waardenburg syndrome | *EDNRB* | | glycogen storage disease type 1A | *G6PC* | | limb-girdle muscular dystrophy-7 (LGMDR7) | *TCAP* | | limb-girdle muscular dystrophy-18 (LGMDR18) | *TRAPPC11* | | cystic fibrosis | *CFTR* | | phenylketonuria | *PAH* | | 6-pyruvoyltetrahydropterin synthase deficiency | *PTS* | | MYH-associated polyposis | *MUTYH* | | hereditary factor XI deficiency disease | *F11* | | hereditary spastic paraplegia type 5 | *CYP7B1* | | hereditary spastic paraplegia type 15 | *ZFYVE26* | | ABCA4-Related Disorders (cone-rod dystrophy/Stargardt macular degeneration) | *ABCA4* | | autosomal recessive bestrophinopathy | *BEST1* | | Cone-rod dystrophy and hearing loss 1 (CRDHL1) | *CEP78* | | Nonsyndromic hearing loss and deafness | *GJB2* | | Pendred syndrome | *SLC26A4* | | Nagashima-type palmoplantar keratosis (NPPK) | *SERPINB7* | | mucolipidosis type III | *GNPTAB* | | glycogen storage disease type II | *GAA* | | **(C)**  Pharmacogenetic associations for drug safety | Lidocaine and Prilocaine | *G6PD* | | Ropivacaine | *G6PD* | | Celecoxib | *CYP2C9* | | Flurbiprofen | *CYP2C9* | | Ibuprofen | *CYP2C9* | | lornoxicam | *CYP2C9* | | meloxicam | *CYP2C9* | | Piroxicam | *CYP2C9* | | Sulfasalazine | *G6PD*, *NAT2* | | Clopidogrel | *CYP2C19* | | Simvastatin | *SLCO1B1* | | Glimepiride | *G6PD* | | Glipizide | *G6PD* | | Rasburicase | *G6PD* | | Amikacin | *MT-RNR1* | | Ceftriaxone | *G6PD* | | Gentamicin | *MT-RNR1* | | Hydroxychloroquine | *G6PD* | | Isoniazid | *NAT2* | | Efavirenz | *CYP2B6* | | Nalidixic Acid | *G6PD* | | Neomycin | *MT-RNR1* | | Paromomycin | *MT-RNR1* | | Peginterferon Alfa-2a | *IFNL3* | | Peginterferon Alfa-2b | *IFNL3* | | Ribavirin | *IFNL3* | | Sulfamethoxazole and Trimethoprim | *G6PD*, *NAT2* | | Streptomycin | *MT-RNR1* | | Tobramycin | *MT-RNR1* | | Carisoprodol | *CYP2C19* | | Flutamide | *G6PD* | | Irinotecan | *UGT1A1* | | Nilotinib | *UGT1A1* | | Mercaptopurine | *TPMT* | | Pazopanib | *UGT1A1* | | Citalopram | *CYP2C19* | | Escitalopram | *CYP2C19* | | Sertraline | *CYP2C19* | | Clobazam | *CYP2C19* | | Azathioprine | *TPMT* | | Tacrolimus | *CYP3A5* | | Methylene Blue | *G6PD* | | Sodium Nitrite | *G6PD* | |
| --- | --- | --- | --- | --- | --- | --- | --- | --- | --- | --- | --- | --- | --- | --- | --- | --- | --- | --- | --- | --- | --- | --- | --- | --- | --- | --- | --- | --- | --- | --- | --- | --- | --- | --- | --- | --- | --- | --- | --- | --- | --- | --- | --- | --- | --- | --- | --- | --- | --- | --- | --- | --- | --- | --- | --- | --- | --- | --- | --- | --- | --- | --- | --- | --- | --- | --- | --- | --- | --- | --- | --- | --- | --- | --- | --- | --- | --- | --- | --- | --- | --- | --- | --- | --- | --- | --- | --- | --- | --- | --- | --- | --- | --- | --- | --- | --- | --- | --- | --- | --- | --- | --- | --- | --- | --- | --- | --- | --- | --- | --- | --- | --- | --- | --- | --- | --- | --- | --- | --- | --- | --- | --- | --- | --- | --- | --- | --- | --- | --- | --- | --- | --- | --- | --- | --- | --- | --- | --- | --- | --- | --- | --- | --- | --- | --- | --- | --- | --- | --- | --- | --- | --- | --- | --- | --- | --- | --- | --- | --- | --- | --- | --- | --- | --- | --- | --- | --- | --- | --- | --- | --- | --- |

**Supplemental Data**

**Data S1. Timeline and milestone of TPMI.**

(Refer to Excel file)

**Data S2. Shared SNPs in TPMv1 and TPMv2 arrays.**

(Refer to Excel file)

**Data S3. Population structure of Taiwan’s indigenous groups.**

(Refer to Excel file)

**Data S4. Genetic admixture analysis using ADMIXTURE–Cross-validation error across different *K* values.**

(Refer to Excel file)

**Data S5. Genetic admixture analysis using ADMIXTURE–Incremental improvements in log-likelihood across different *K* values.**

(Refer to Excel file)

**Data S6. Genetic admixture analysis using ADMIXTURE–Hierarchical clustering dendrogram of the Fst matrix.**

(Refer to Excel file)

**Data S7. Homozygosity analysis–Heatmap of homozygosity rate.**

(Refer to Excel file)

**Data S8. Homozygosity analysis–Violin plots of homozygosity rate.**

(Refer to Excel file)

**Data S9. Sample size evaluation and examples for GWAS and QTL mapping–Sample size calculation for a case-control study.**

(Refer to Excel file)

**Data S10. Sample size evaluation and examples for GWAS and QTL mappings–Sample size calculation for quantitative trait locus (QTL) study.**

(Refer to Excel file)

**Data S11. Sample size evaluation and examples for GWAS and QTL mapping–Miami plot of the GWAS for Type 2 Diabetes (T2D) and QTL mapping for HbA1c.**

(Refer to Excel file)

**Data S12. Manhattan plots for a GWAS of Essential Hypertension and two blood pressures–Essential Hypertension (EHT, 71,548 cases and 130,561 controls).**

(Refer to Excel file)

**Data S13. Manhattan plots for a GWAS of Essential Hypertension and two blood pressures–Systolic blood pressure (SBP, n = 241,667).**

(Refer to Excel file)

**Data S14. Manhattan plots for a GWAS of Essential Hypertension and two blood pressures–Diastolic blood pressure (DBP, n = 241,646).**

(Refer to Excel file)

**Data S15. Sensitivity analyses considering different SBP cutoffs for a GWAS of Essential Hypertension–Essential Hypertension: Definition 2 (EHT defined by the ICD10-code I10, SBP >=130 mmHg, or DBP >=80 mmHg; 70,884 cases and 149,639 controls)**

(Refer to Excel file)

**Data S16. Sensitivity analyses considering different SBP cutoffs for a GWAS of Essential Hypertension–Essential Hypertension: Definition 3 (EHT defined by the ICD10-code I10, SBP >=140 mmHg, or DBP >=80 mmHg; 68,788 cases and 162,337 controls)**

(Refer to Excel file)

**Data S17. Detailed GWAS results.** In addition to SNP information (Chromosome, physical position, SNP rs ID, and two alleles), this table provides risk allele (EA), beta value (Beta), p-value (P), and rank of association signals (Rank) for each biobank. In addition to TPMI, GWAS summary statistics from the PheWebs of the Biobank Japan (BBJ), China Kadoorie Biobank (CKB), Korean Genome and Epidemiology Study (KoGES), and UK Biobank (UKB) are provided, if available. Novel association signals identified by the TPMI are marked in red (studied biobanks do not include this SNP) or orange (studied biobanks have an insignificant result, with p-value > Bonferroni’s level). The genome-wide significance levels are 10^-8^, 3.80×10^-9^, 5.73×10^-9^, 6.26×10^-9^, and 4.59×10^-9^ for TPMI, BBJ, CKB, KoGES, and UKB, respectively.

(Refer to Excel file)

**Data S18. Citation for the significant SNPs identified by the TPMI and replicated in other biobank studies.** In addition to SNP information (SNP RSID, AffyID, and p-value (p)), this table includes the GWAS Catalog ID, trait name, sample size, PubMed ID, Published journal, and article title. SNPs reported in the literature as T2D-associated are marked in red.

(Refer to Excel file)

**Data S19. Functional annotation of novel association signals identified in our GWAS for T2D.** This table includes annotation information from columns A to AH. Each variable name and its corresponding description are provided, followed by the detailed annotation data.

(Refer to Excel file)

**Data S20. Gene set enrichment and pathway analysis for novel SNP-associated genes from our T2D GWAS using Ingenuity Pathway Analysis (IPA).**

(Refer to Excel file)

**Data S21. Manhattan plots for REGENIE analysis of T2D, HbA1c, EHT, and two blood pressures (SBP and DBP)–GWAS of Type 2 Diabetes (T2D) (n = 52,290 cases and 192,817 controls).**

(Refer to Excel file)

**Data S22. Manhattan plots for REGENIE analysis of T2D, HbA1c, EHT, and two blood pressures (SBP and DBP)–QTL mapping of Hemoglobin A1c (HbA1c) (n = 140,259).**

(Refer to Excel file)

**Data S23. Manhattan plots for REGENIE analysis of T2D, HbA1c, EHT, and two blood pressures (SBP and DBP)–GWAS of Essential Hypertension (EHT) (n = 71,548 cases and 130,561 controls).**

(Refer to Excel file)

**Data S24. Manhattan plots for REGENIE analysis of T2D, HbA1c, EHT, and two blood pressures (SBP and DBP)–QTL mapping of systolic blood pressure (SBP) (n = 241,667).**

(Refer to Excel file)

**Data S25. Manhattan plots for RFGENIE analysis of T2D, HbA1c, EHT, and two blood pressures (SBP and DBP)–QTL mapping of diastolic blood pressure (DBP) (n = 241,646).**

(Refer to Excel file)

**Data S26. Polygenic risk score analysis based on T2D PGS (PGS002308)–Area under the receiver operating characteristic curve (AUC).**

(Refer to Excel file)

**Data S27. Polygenic risk score analysis based on T2D PGS (PGS002308)–Dose-response effect of PRS levels on the odds ratio of T2D.**

(Refer to Excel file)

**References**
