# Supplementary material for: The Taiwan Precision Medicine Initiative provides a cohort for large-scale studies
Source: Nature. 2025 Oct 15;648(8092):117–27. doi: 10.1038/s41586-025-09680-x (PMC12675286; doi:10.1038/s41586-025-09680-x)
Supplement: Supplementary file 2 — Reporting Summary [file 41586_2025_9680_MOESM2_ESM.pdf]

nature portfolio

Corresponding author(s): Pui-Yan Kwok

Last updated by author(s): Sep/12/25

Reporting Summary

Nature Portfolio wishes to improve the reproducibility of the work that we publish. This form provides structure for consistency and transparency in reporting. For further information on Nature Portfolio policies, see our Editorial Policies and the Editorial Policy Checklist.

Statistics

For all statistical analyses, confirm that the following items are present in the figure legend, table legend, main text, or Methods section.

n/a	Confirmed	
☐	☒	The exact sample size (n) for each experimental group/condition, given as a discrete number and unit of measurement
☐	☒	A statement on whether measurements were taken from distinct samples or whether the same sample was measured repeatedly
☐	☒	The statistical test(s) used AND whether they are one- or two-sided<br>*Only common tests should be described solely by name; describe more complex techniques in the Methods section.*
☐	☒	A description of all covariates tested
☐	☒	A description of any assumptions or corrections, such as tests of normality and adjustment for multiple comparisons
☐	☒	A full description of the statistical parameters including central tendency (e.g. means) or other basic estimates (e.g. regression coefficient) AND variation (e.g. standard deviation) or associated estimates of uncertainty (e.g. confidence intervals)
☐	☒	For null hypothesis testing, the test statistic (e.g. F, t, r) with confidence intervals, effect sizes, degrees of freedom and P value noted<br>*Give P values as exact values whenever suitable.*
☒	☐	For Bayesian analysis, information on the choice of priors and Markov chain Monte Carlo settings
☒	☐	For hierarchical and complex designs, identification of the appropriate level for tests and full reporting of outcomes
☒	☐	Estimates of effect sizes (e.g. Cohen's d, Pearson's r), indicating how they were calculated

Our web collection on statistics for biologists contains articles on many of the points above.

Software and code

Policy information about availability of computer code

Data collection	Electronic medical record (EMR) data, including both free text and structured data, were extracted from the partner medical centers by using a comprehensive suite of tools. The extraction process involved the application of self-developed regular expressions, self-trained nature language models, and the PostgreSQL and SpaCy stack (python v3.9.6 and spaCy v3.5.0). To ensure privacy and data protection, personal names were dynamically detected and removed using the CKIP NLP stack toolkit, running on a CentOS v7.9.2009 environment, with python v3.6.8, ckiptagger v0.2.1, and TensorFlow v1.13.1. Genotype calling data was performed using Applied Biosystems™ Array Power Tools (APT), with APT v2.10.2.2 applied for TPMv1 SNP array data and APT v2.11.3 for TPMv2 SNP array data.
Data analysis	In imputation analysis, alignment was done by using BWA, variant calling was done by using DeepVariant, read-based phasing was done with WhatsHap, and haplotype phasing and genome imputation were done with SHAPEIT4 and IMPUTE5; Familial relativeness analysis was carried out by using KING 2.2.7; Population structure analysis and principal component analysis were conducted by using PLINK 2.0; Homozygosity analysis was carried out by using PLINK 2.0; In admixture analysis, genetic ancestry fractions were estimated using ADMIXTURE 1.3.0; Sample size was evaluated by using QUANTO 1.2.4; Quality control was carried out by using PLINK in cooperation with KING and R; Genome-wide association study was conducted by using PLINK 2.0 and REGENIE 4.1; Multi-ancestry PRS was constructed by using a Python based command line tool PRS-CSx.<br><br>The code used for data analysis and quality control is available at https://github.com/HsinChouYang/TPMI_Cohort/.

For manuscripts utilizing custom algorithms or software that are central to the research but not yet described in published literature, software must be made available to editors and reviewers. We strongly encourage code deposition in a community repository (e.g. GitHub). See the Nature Portfolio guidelines for submitting code & software for further information.

Data

Policy information about availability of data

All manuscripts must include a data availability statement. This statement should provide the following information, where applicable:

- Accession codes, unique identifiers, or web links for publicly available datasets
- A description of any restrictions on data availability
- For clinical datasets or third party data, please ensure that the statement adheres to our policy

Data Availability statement:
All summary statistics and results from this study are freely available from the TPMI website (https://tpmi.ibms.sinica.edu.tw). In compliance with the data protection laws governing genetic and health data in Taiwan, the de-identified TPMI clinical and genotyping data are kept in a secure server at the Academia Sinica and not released to the public. TPMI is in the process of transitioning to a biobank model, which will make the TPMI data openly accessible to the public for research purposes by December 2026, and data analysis will be performed on local servers in a trusted research environment. Researchers who wish to access the individual clinical and genotyping data prior to that time can do so by collaboration through a 4-step process: 1. Complete an on-line application form posted on the TPMI website with specific proposals for collaboration; 2. The TPMI Feasibility Committee will assess the scientific, clinical, technical, resource, and regulatory feasibility of the proposals at a monthly meeting and approve all feasible proposals, with priority given to those aligned with TPMI's mission, resource capacity, and compliance obligations; 3. A TPMI team will work with the applicant of the approved proposal to prepare a protocol of the project for review by the Academia Sinica Institutional Review Board (IRB); 4. Once the IRB approval is obtained, the collaborative work will be performed by the TPMI team according to the collaborators' proposed study design. Summary statistics and analysis results will be delivered to the collaborators. To initiate a collaboration, please contact the TPMI team at https://tpmi.ibms.sinica.edu.tw/index.html

In addition to the TPMI data, we analyzed the following external datasets as part of the validation:
- Meta-GWAS summary statistics for T2D across multiple populations from the DIAGRAM Consortium are available at https://diagram-consortium.org/downloads.html.
- The linkage disequilibrium reference from various populations of the 1000 Genomes Project can be downloaded from https://github.com/getian107/PRScsx.
- PRS-CSx weights for T2D across multiple populations from the PGS Catalog are available at https://www.pgscatalog.org/score/PGS002308/.
- The 1000 Genomes Project data can be accessed via PLINK 2.0 at https://www.cog-genomics.org/plink/2.0/resources#phase3_1kg.
- Fastq files of Simons Genome Diversity Project (SGDP) samples are available at https://www.internationalgenome.org/data-portal/data-collection/sgdp.
- Genotype data from the Taiwan Biobank are available through a formal application process (https://www.twbiobank.org.tw/index.php).
- Biobank Japan (BBJ) GWAS summary statistics can be assessed through the BioBank Japan PheWeb (https://pheweb.jp)
- China Kadoorie Biobank (CKB) GWAS summary statistics can be assessed through the China Kadoorie Biobank PheWeb (https://pheweb.ckbiobank.org)
- Korean Genome and Epidemiology Study (KoGES) GWAS summary statistics can be assessed through the KoGES PheWeb (https://koges.leelabsg.org)
- UK Biobank (UKB) GWAS summary statistics can be assessed through the UKBiobank PheWeb (https://pheweb.org/UKB-Neale)

Research involving human participants, their data, or biological material

Policy information about studies with human participants or human data. See also policy information about sex, gender (identity/presentation), and sexual orientation and race, ethnicity and racism.

Reporting on sex and gender

Analyses and reports for males and females are included.

Reporting on race, ethnicity, or other socially relevant groupings

The population structure of the TPMI cohort was assessed against external resources with known population information from various genetic projects, including the TWB, the Simons Genome Diversity Project (SGDP), and the 1000 Genomes Project (1KG). The TWB dataset encompassed 83,664 individuals, consisting of 68,023 with Minnan ancestry, 11,549 with Hakka ancestry, and 4,092 Han Mainlanders, further categorized into 1,681 Southern Han, 1,606 Central Han, and 805 Northern Han based on self-reported birth geographic regions. The SGDP dataset included 3 individuals from two Taiwan indigenous tribes, namely 1 Atayal people and 2 Ami people, to assess the genetic contribution of indigenous populations in Taiwan. Within the 1KG dataset, there were 3,202 individuals representing 26 global populations across five continents – Africa (AFR), Americas (AMR), East-Asia (EAS), South-Asia (SAS), and Europe (EUR). This dataset comprised 893 with AFR ancestry, 490 with AMR ancestry, 585 with EAS ancestry, 601 with SAS ancestry, and 633 with EUR ancestry. The EAS-ancestry group consisted of 104 Japanese in Tokyo, Japan (JPT), 103 Han Chinese in Beijing, China (CHB), 163 Southern Han Chinese, China (CHS), 93 Chinese Dai in Xishuangbanna, China (CDX), and 122 Kinh in Ho Chi Minh City, Vietnam (KHV). Reporting about population structure has been provided.

Population characteristics

The main focus in this paper is the Han Chinese population in Taiwan. Among the 486,956 participants with both genotype and EMR data in the TPMI cohort, there are 217,595 male participants with an average age of 57.4 (standard deviation = 17.5) and 269,361 female participants with an average age of 54.9 (standard deviation = 17.0). The majority of participants fall within the age range of 20 to 90, with over 160 individuals aged over 100.

Recruitment

To ensure compliance with local guidelines, we have followed the Taiwan Ministry of Health and Welfare (MOHW) regulations outlined for ethical approval, patient data protection, and clinical research and care. Participants were recruited from 16 partner medical centers (encompassing 33 affiliated hospitals) that together serve ~40% of the population in Taiwan (Fig. 1). On-site physicians and nurses facilitated the enrollment process. Informed consent was obtained from the participants while they were enrolled in this study at the hospitals. After providing informed consent, participants donated blood samples for genotyping and agreed to have their electronic medical records (EMR) de-identified, encrypted, and securely transmitted to the TPMI server. Participants' genetic profiles were conducted using two customized TPMI SNP arrays (TPMv1 and TPMv2). Participation was offered to all except for those whose peripheral blood cells might harbor non-germline genetic materials: (a) individuals with leukemia who did not achieve remission; (b) individuals who received blood transfusions within the previous six months; (c) individuals who underwent chemotherapy or radiotherapy within the previous 12 months. As of Dec 28, 2023 (TPMI v37 data freeze), 565,390 participants had been enrolled with proper consent.

Before inviting patients or health check-up participants to join TPMI, physicians, nurses, or research staff at affiliated hospitals explained the informed consent form to ensure participants' understanding. It was emphasized that participation was entirely voluntary and would not affect their medical rights, regardless of their decision. Participants were also informed that they could withdraw from the study at any time. Participants proceeded with samples and data collection only after signing the informed consent form.

The recruitment process in the TPMI study may be subject to some degree of self-selection bias (in other words, individuals who are more health-conscious or already receiving medical care may be more likely to participate) and other potential biases (such as age and health-status bias, geographic bias, and ethnic and socioeconomic bias), we have made substantial efforts to recruit a diverse and representative sample. We also used advanced statistical techniques to account for these biases in our analyses. Despite these challenges, the TPMI study provides valuable insights into precision medicine, and we believe the findings are both robust and relevant to the broader Taiwanese population.

Ethics oversight

This study was approved by the Institutional Review Boards of Taipei Veterans General Hospital (2020-08-014A), National Taiwan University Hospital (201912110RINC), Tri-Service General Hospital (2-108-05-038), Chang Gung Memorial Hospital (201901731A3), Taipei Medical University Healthcare System (N202001037), Chung Shan Medical University Hospital (CS19035), Taichung Veterans General Hospital (SF19153A), Changhua Christian Hospital (190713), Kaohsiung Medical University Chung-Ho Memorial Hospital (KMUHIRB-SV(II)-20190059), Hualien Tzu Chi Hospital (IRB108-123-A), Far Eastern Memorial Hospital (110073-F), Ditmanson Medical Foundation Chia-Yi Christian Hospital (IRB2021128), Taipei City Hospital (TCHIRB-10912016), Koo Foundation Sun Yat-Sen Cancer Center (20190823A)
Cathay General Hospital (CGH-P110041), Fu Jen Catholic University Hospital (FJUH109001)
and Academia Sinica (AS-IRB01-18079), Taiwan. Written informed consent was obtained from the subjects in accordance with institutional requirements and the Declaration of Helsinki principles. All collected information was de-identified before statistical data analysis.

Note that full information on the approval of the study protocol must also be provided in the manuscript.

Field-specific reporting

Please select the one below that is the best fit for your research. If you are not sure, read the appropriate sections before making your selection.

☒ Life sciences ☐ Behavioural & social sciences ☐ Ecological, evolutionary & environmental sciences

For a reference copy of the document with all sections, see nature.com/documents/nr-reporting-summary-flat.pdf

Life sciences study design

All studies must disclose on these points even when the disclosure is negative.

Sample size

The sample size for the Taiwan Precision Medicine Initiative (TPMI) was not derived from a formal single-hypothesis power calculation, but was instead set strategically, in line with other landmark precision medicine cohorts, such as the UK Biobank (500,000 participants) and the U.S. All of Us Research Program (1 million participants). For TPMI, the milestone of 500,000 participants with whole-genome genotyping and electronic medical records (EMR) data was chosen based on the following rationale: (1) Global relevance: To establish a globally significant reference cohort for precision medicine, particularly addressing the underrepresentation of East Asian populations in biobank-scale research. (2) Statistical power and prediction accuracy: To ensure adequate statistical power and prediction accuracy across a wide spectrum of complex diseases and quantitative traits relevant to the Han Chinese population. (3) Variant detection: To enable the discovery of both common and low-frequency variants with modest effect sizes. (4) Subgroup Analyses: To facilitate stratified analyses across sex, age, and environmental exposures. (5) Longitudinal insights: To support prospective follow-up studies of disease trajectories, treatment responses, and gene–environment interactions through EMR linkage.

Data exclusions

Our analysis only removed the participants and SNPs which were failed in the standard quality control check of a genetic study.

Replication

To ensure the reproducibility and robustness of our findings, we implemented several key measures during the study: (1) Replication strategy: As part of our quality control and validation process, we compared the results of our GWASs for various diseases and quantitative traits in the TPMI cohort with publicly available datasets, including Biobank Japan (BBJ), China Kadoorie Biobank (CKB), Korean Genome and Epidemiology Study (KoGES), and UK Biobank (UKB). This allowed us to assess the replicability of the associations we identified and to verify the consistency of findings across different populations. (2) Successful replication: We observed that several genetic associations identified in the TPMI GWAS were also found in the BBJ, CKB, KoGES, and UKB, demonstrating consistent replication of key results across these large, independent cohorts (Supplementary Data 1). This consistency supports the reliability and generalizability of our findings. (3) Novel associations: In addition to replicating previously identified associations, our study also uncovered novel genetic associations that had not been identified by the other biobanks (Supplementary Data 3). These findings provide new insights into the genetic basis of the diseases and traits studied, highlighting the value of the TPMI cohort in contributing to the broader understanding of human genetics. (4) Challenges and limitations: Although we were able to replicate a significant number of associations, some findings were not replicated across all cohorts. This is expected in genetic research, as differences in study design, sample sizes, and population demographics (e.g., ethnic background) can influence the reproducibility of specific genetic signals. For the associations that were not replicated, the potential reasons include population-specific effects, environmental factors, or differences in statistical power and sample size between the cohorts.

Randomization

In the context of the Taiwan Precision Medicine Initiative (TPMI), randomization is generally not necessary for the studies since TPMI is primarily based on observational research rather than experimental clinical trials.

Blinding

At the current stage, the Taiwan Precision Medicine Initiative (TPMI) study is an observational study, not a clinical trial. Therefore, the concept

Blinding	of blinding, which is typically used in clinical trials to reduce bias in outcomes, does not apply to our study design.

Reporting for specific materials, systems and methods

We require information from authors about some types of materials, experimental systems and methods used in many studies. Here, indicate whether each material, system or method listed is relevant to your study. If you are not sure if a list item applies to your research, read the appropriate section before selecting a response.

Materials & experimental systems

n/a	Involved in the study
☒	☐ Antibodies
☒	☐ Eukaryotic cell lines
☒	☐ Palaeontology and archaeology
☒	☐ Animals and other organisms
☒	☐ Clinical data
☒	☐ Dual use research of concern
☒	☐ Plants

Methods

n/a	Involved in the study
☒	☐ ChIP-seq
☒	☐ Flow cytometry
☒	☐ MRI-based neuroimaging

Plants

Seed stocks	Not available
Novel plant genotypes	Not available
Authentication	Not available
